# A deep neural network approach for optimizing charging behavior for electric vehicle ride-hailing fleet

**DOI:** 10.1038/s41598-025-05953-7

**Published:** 2025-07-01

**Authors:** Kaizhe Chen, Jin Liu, Wenjing Lyu, Tianyuan Wang, Jinxi Wen

**Affiliations:** 1 School of Education, Beijing Institute of Technology, Beijing, China; 2 Computer Science and Artificial Intelligence Laboratory, Massachusetts Institute of Technology Cambridge, American, United States; 3 School of Management, Zhejiang University, Zhejiang, China

**Keywords:** Climate-change mitigation, Climate-change policy, Energy and society

## Abstract

The rapid advancement of Artificial Intelligence (AI) has led to a profound transformation in the transportation industry, particularly in driving the shift toward carbon neutrality and electrification. AI has proven to be a key enabler in formulating innovative strategies for optimizing electric vehicle (EV) fleets, thus advancing transportation services. While extensive research has been conducted on AI’s role in transportation innovation, there remains a significant gap in empirical studies focusing on optimizing the charging behavior of operational EV fleets, particularly within ride-hailing services. The rise of ride-hailing services has revolutionized the transportation landscape, and their transition to EV fleets presents a major opportunity. The integration of AI to optimize the operations of these EV ride-hailing fleets could substantially help achieve the dual objectives of reducing charging costs and simultaneously lowering carbon emissions. Therefore, this research develops a Neural Network (NN) trained with the Adaptive Moment Estimation (Adam) algorithm, based on 2.14 million charging events. The goal is to analyze current charging behaviors and evaluate the impact of key variables on costs and emissions, providing data-driven insights for potential improvements, thus addressing a critical research gap. The novelty of this study lies in its novel combination of deep learning algorithms with large-scale real-world charging data, proposing a new method for optimizing EV ride-hailing charging behavior, and providing practical solutions for promoting electric vehicle adoption and achieving low-carbon transportation.

**Supplementary Information:**

The online version contains supplementary material available at 10.1038/s41598-025-05953-7.

## Introduction

Driven by the dual forces of energy and digital revolution, the global transportation sector is undergoing a green and low-carbon transformation, with energy technology innovation reaching a period of sustained high activity^1,2^. Faced with increasingly severe challenges such as energy resource constraints, ecological degradation, and escalating climate change, countries worldwide are accelerating their pace toward decarbonization. This has led to a shift both in the energy industry and transportation sector towards technology-intensive, significantly impacting the world’s geopolitical landscape and socioeconomic progress^3–7^.

With this backdrop, the integration of cutting-edge information technologies, such as Big Data, Cloud Computing, the Internet of Things (IoT), Artificial Intelligence (AI), and Blockchain, has been transformative. Specifically in the transportation sector, these technologies have synergized with traditional energy technologies, fostering innovative models and formats^8–10^. Indeed, the advent of AI-driven transportation innovation is becoming pivotal in realizing sustainable development goals. To alleviate the strain on the power grid, conventional approaches such as grid expansion and reinforcement of grid components are available, albeit entailing complexities and high costs in implementation^11^. The more intelligent and cost-effective approach involves the utilization of emerging technologies to optimize the charging infrastructure and drivers’ charging strategies. The progress in AI has introduced novel technological paradigms, including Vehicle-to-Grid (V2G), Internet of Energy (IoE), and Internet of Electric Vehicles (IoEV). These concepts have provided updated and high-quality solutions for charging management. For instance, machine learning or AI algorithms can predict driver behavior and select efficient driving routes^12–15^. Moreover, they can be utilized to improve battery thermal management^16,17^. Sugii et al. (1999) proposed a genetic algorithm-based scheduling method for charging multiple electric vehicles^18^. This method can determine power curves and leverage power load, thereby reducing charging equipment capacity and initial costs. Panahi et al. (2015) suggested using artificial neural networks to predict daily load curves for individual electric vehicles and vehicle fleets^19^. Specifically, they utilized historical data to forecast power demand and achieve better coordination of charging. Sachan et al. (2020) compared three different charging infrastructures based on various factors, such as EV availability, driving patterns, charging time, return time from the last trip, charging status, battery capacity, and plug availability^20^. The simulation results demonstrated the advantages of intelligent charging over dumb charging strategies. These studies highlight the potential of intelligent charging solutions, utilizing techniques like genetic algorithms and artificial neural networks, to optimize charging strategies for multiple electric vehicles and improve the overall efficiency and cost-effectiveness of the charging process. Of notable significance is the role of AI in optimizing the operation of Electric Vehicle (EV) fleets, specifically ride-hailing services, which represent a major shift in transportation innovation^21^. The efficient and strategic use of AI can optimize EV charging behaviors, minimizing costs and reducing carbon emissions concurrently.

The worldwide growth of EVs further indicates a progressive move towards greener transportation^22^. Governments have implemented various incentives to encourage the advancement and adoption of EVs^23^such as reducing purchase costs, investing in technological research and innovation, and the development of charging infrastructure. This has led to an increasing adoption rate and market penetration of EVs globally^2,24,25^. Take China as an example. As the world’s largest energy producer, consumer, and carbon emitter for several consecutive years, it faces significant challenges in achieving green development and energy transition. To this end, China has instituted measures to promote the development of electrified transportation, aiming for new energy vehicles to make up about 20% of total new car sales by 2025. Meanwhile, China is also actively investing in optimizing the layout of charging infrastructure, promoting coordinated operations of EVs and charging facilities, and advancing bidirectional energy and information interaction between EVs and smart grids (*Global EV Outlook 2020 – Analysis*, 2020).

This study is motivated by the global shift towards sustainable transportation and the critical role of AI in this endeavor. It aims to address existing gaps in the literature and provide valuable insights into developing effective AI models for guiding EV ride-hailing operators to optimize charging behavior. By doing so, it anticipates contributing to the broader goals of energy security, industrial advancement, and environmental preservation.

AI-based optimization algorithms play a crucial role in deriving charging solutions based on specific optimization objectives. These include minimizing charging costs, minimizing power losses, ensuring voltage stability, reducing peak loads, reducing queues at charging stations, optimizing EV routes, and mitigating line, grid, and transformer overloads. Previous literature has utilized various optimization techniques, including mathematical optimization, heuristic algorithms, Particle Swarm Optimization (PSO), Genetic Algorithms (GA), and fuzzy logic^27–31^. Given the multi-objective nature of EV charging solutions, AI allows for the application of different algorithms to find near-optimal solutions for specific charging scenarios. The choice of optimization technique depends on the complexity of the problem and the particular objectives at hand. Therefore, the selection of optimal EV charging strategies hinges on the appropriate choice of optimization objectives and AI-driven techniques.

While a wealth of research exists on the role of AI in transportation innovation, particularly in optimizing EV fleets, there is a distinct scarcity of empirical studies that specifically address the optimal charging behavior of operational EV fleets, notably within the context of ride-hailing services. These studies would ideally target the dual-objective of minimizing charging costs and reducing carbon emissions concurrently. As a significant paradigm shift in recent transportation innovation, ride-hailing services harbor immense potential towards achieving carbon neutrality, particularly when synergized with AI-guided logistical optimizations. Therefore, the development of AI models capable of providing strategic guidance for EV ride-hailing operators to optimize their charging behaviors is a research area ripe for exploration.

Grounded in the real-world challenges that EV ride-hailing fleets encounter, our study aims to optimize the charging strategies at the fleet level, with the dual goal of minimizing charging costs and reducing carbon emissions across the entire fleet. Utilizing a Neural Network (NN) trained with the Adaptive Moment Estimation (Adam) algorithm, our research attempts to address the following fleet-level queries:

1. What is the optimal time to initiate charging for the entire fleet to reduce both charging costs and carbon emissions?
2. What should be the optimal initial State of Charge (SOC) for the fleet to achieve the aforementioned goals?
3. What is the optimal charging speed for the fleet to reduce charging costs and carbon emissions?
4. Should EV ride-hailing operators strategically select charging facilities for the fleet?

Specifically, this study matched nearly 2.14 million charging events from approximately 10,000 electric vehicle ride-hailing fleets in Beijing, collected from January 2019 to October 2020, with a geospatial digital map. This allowed us to identify the 11,214 residential communities nearest to these charging events, thereby categorizing the charging as home or non-home-based. Subsequently, we estimated charging costs by matching each charging event with real-time grid power prices, and carbon emissions were estimated by considering the subsequent driving event following each charging session. Following this, an Adam algorithm trained Neural Network was deployed to predict the optimized charging costs and carbon emissions. This was based on optimal starting charging times, starting SOC, and charging speed under both home and non-home-based charging scenarios.

It is worth noting that the optimization in this study encompasses two main aspects: one involves minimizing prediction errors in the model using the Adam algorithm to enhance its accuracy in predicting charging costs and carbon emissions; the other involves optimizing the charging behavior of electric vehicle fleets to reduce both costs and emissions. Thus, our approach aims to strike a balance between improving model performance and achieving optimal charging strategies.

This research endeavor provides a significant contribution to the understanding of intelligent charging strategies for ride-hailing EV fleets. It, therefore, offers indispensable insights for the development of effective pricing structures and policies, which can guide EV ride-hailing operators in optimizing their charging behaviors. The pivotal findings of this study suggest that, for these operators, scheduling their charging activities around 15:00 at a charging facility with a power output of 20–40 kW, and ideally initiating the process at a relatively low SOC, could provide an optimal balance between reducing charging costs and minimizing carbon emissions. Moreover, our results underscore that home charging, particularly at speeds above 20 kW, emerges as a cost-effective solution for EV ride-hailing operators.

## Results

### Current charging behavior patterns of electric vehicle ride-hailing fleet

**Fig. 1:**
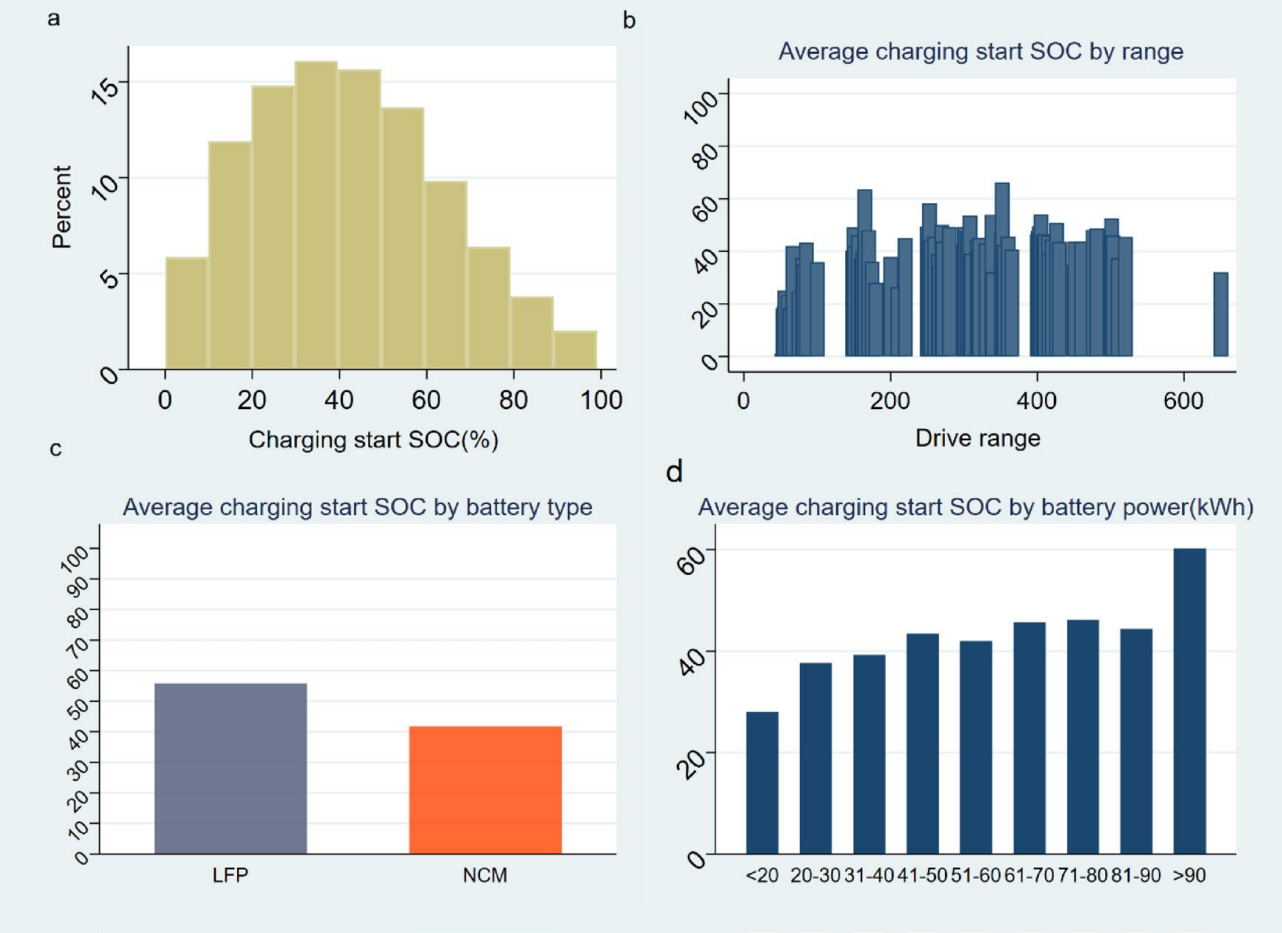
Charging Start SOC and Time| **a**, Distribution of SOC at charging start. Over 60% of events begin when the SOC is between 20% and 60%, with 25% occurring between 30% and 45%. Only 2% of events start below 5% or above 90%. **b**, Average starting SOC by driving range: Most ranges show an average SOC around 40%, except for vehicles with a 170 km range, which start charging at 82% SOC. **c**, Average starting SOC by battery type: LFP batteries initiate charging at an average SOC of 57%, while NCM batteries start at 41%. **d**, Average starting SOC by battery capacity: Vehicles with under 20 kWh batteries start at 28%, while those with over 90 kWh batteries start at 60%.

We first report the distribution of current charging behavior patterns of the electric vehicle ride-hailing fleet. Figure 1a reveals the distribution of the starting State of Charge (SOC) for each charging event. This distribution exhibits a right-skewed, inverted U-shaped pattern. Most of the charging events—over 60% of them—begin when the SOC is between 20% and 60%. More specifically, more than 25% of charging events commence when the SOC is within the range of 30–45%. Interestingly, only a small fraction, around 2%, of charging events start when the SOC is below 5%. Similarly, very few charging events, approximately 2%, occur when the SOC is 90% or more.

Figure 1b unveils the distribution of the average starting SOC with respect to the driving range of various electric vehicles. Interestingly, with one exception, the average starting SOC across most driving ranges is centered approximately around 40%. The anomaly lies with electric vehicles that possess a driving range of 170 km. For these vehicles, the average starting SOC for charging is strikingly high, roughly at 82%. This indicates that drivers of these particular vehicles tend to initiate charging when their battery levels are still relatively high, diverging from the common behavior observed across other driving ranges.

Illuminated in Fig. 1c is the average starting SOC distribution in relation to different types of batteries - specifically, Lithium Iron Phosphate (LFP) and Nickel Cobalt Manganese (NCM) batteries. There’s a noticeable disparity between the average starting SOC of these two battery types. For LFP batteries, the average SOC at the onset of charging is roughly 57%, whereas for NCM batteries, it’s significantly lower at approximately 41%. This difference, surpassing 15%, indicates that vehicles equipped with LFP batteries generally initiate charging at a higher SOC than those using NCM batteries. This could be reflective of different charging behavior patterns driven by the unique characteristics of these battery types.

On average charging start SOC by battery power, there appears a clear trend indicating that larger battery capacities correlate with higher average starting SOCs, as shown in Fig. 1d, for batteries under 20 kWh, the average SOC at the time of charging initiation is 28%. As the battery power increases, specifically for those in the 31–90 kWh range, the average SOC at charging initiation surpasses 40%. Remarkably, batteries over 90 kWh begin charging at an even higher average SOC of approximately 60%. This suggests that drivers of vehicles with larger battery capacities tend to wait until their batteries are relatively full before initiating a charge.

**Fig. 2:**
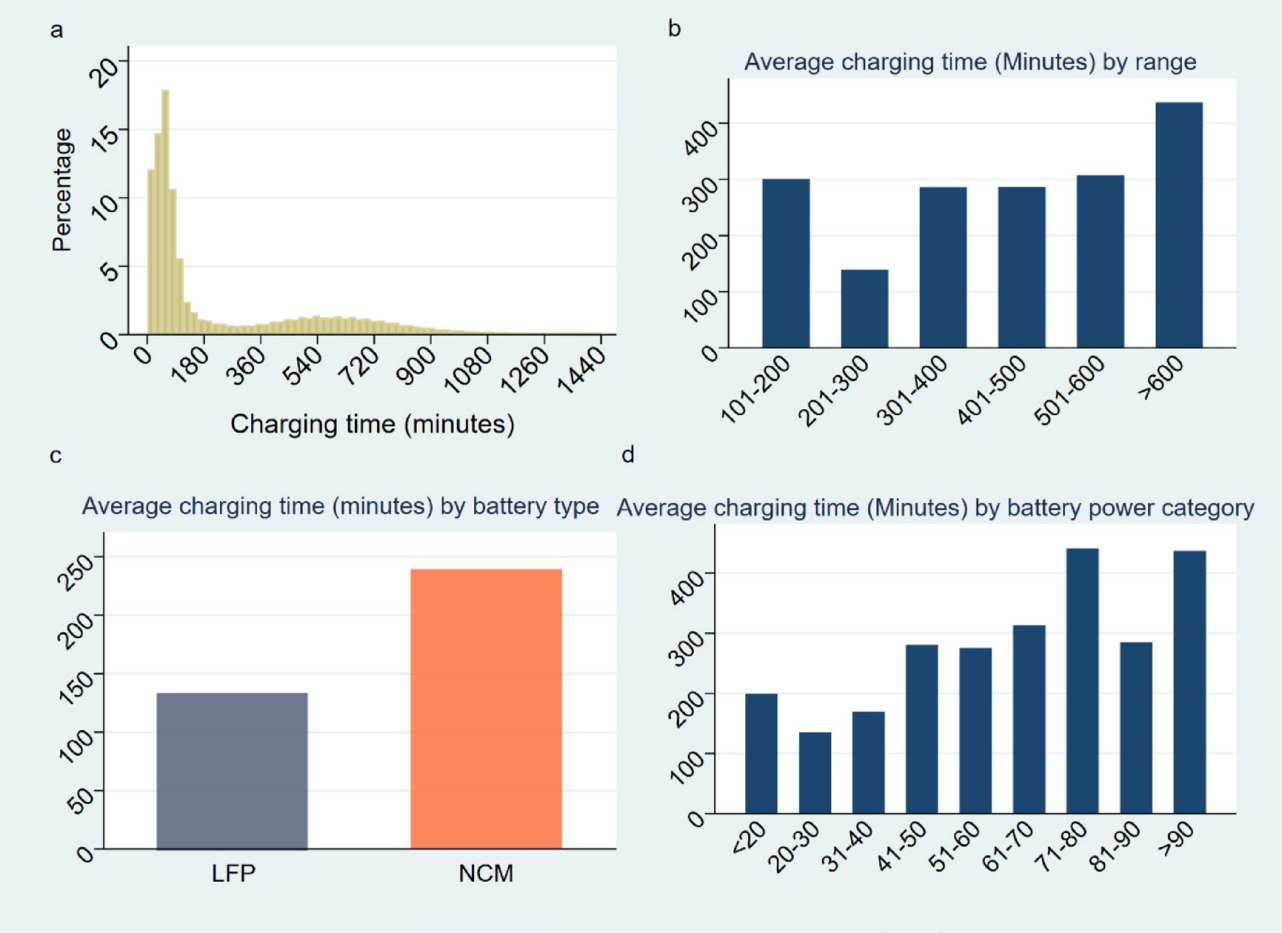
Charging Time| **a**, Distribution of charging durations, with over 60% of events lasting within 2 h and a gradual increase in duration from 300 to 600 min, suggesting that some events require longer times due to factors like charging rate and battery capacity. **b**, Charging time by driving range. Vehicles with a range < 100 km take the longest to charge (540 min), while those with 200–600 km range charge in 220–300 min. Vehicles > 600 km take 420 min. **c**, Charging duration by battery type. LFP batteries charge in 140 min, while NCM batteries take 240 min, due to differing electrochemical properties. **d**, Charging time by battery capacity. Larger batteries take longer to charge. 20–30 kWh batteries charge in 130 min, while 71–80 kWh and > 90 kWh batteries take 440 min.

Figure 2a discloses the distribution of charging durations for the electric vehicle ride-hailing fleet. As shown in Fig. 2a, a significant majority of charging events (over 60%) have durations that fall within a 2-hour window. Specifically, charging events that last about an hour account for nearly 18% of all occurrences. Additionally, an observable, albeit gradual, increase in frequency is detected in the charging duration distribution from roughly 300 min to 600 min. This observation could suggest that while most charging events are relatively short, there are still a significant number of charging sessions that require longer periods, perhaps due to factors such as lower charging rates, higher initial SOC, or larger battery capacities. Figure 2b presents the average charging time of electric vehicles categorized by driving range. Vehicles with a driving range of less than 100 km exhibit the longest charging times, averaging over 540 min. In contrast, those within the 100 km to 200 km range have an average charging time of approximately 400 min. For vehicles with driving ranges between 200 km and 600 km, the charging time decreases progressively, ranging from 220 to 300 min. Notably, vehicles with a range exceeding 600 km experience an increase in charging time, reaching approximately 420 min. This trend indicates that smaller vehicles with shorter ranges generally require longer charging durations, while those with medium to large ranges display more consistent and lower charging times.

**In** Fig. 2c, the relationship is depicted between average charging duration and battery type. From this graph, it is evident that the two battery types - LFP and NCM - exhibit notable differences in their charging durations. LFP batteries display an expedited charging process, with the average duration amounting to roughly 140 min. This is significantly shorter when compared to NCM batteries, which require a considerably lengthier charging time, averaging nearly 240 min. This divergence in charging time can be attributed to the different electrochemical properties of these two types of batteries, influencing their charging characteristics and efficiency. Consequently, the type of battery utilized in an electric vehicle can significantly impact its operational efficiency and, in turn, the efficacy of ride-hailing services employing such vehicles. Figure 2d showcases the correlation between average charging duration and the battery’s power capacity. As one can observe, the average charging duration generally exhibits an upward trend along with an increase in battery capacity. This trend suggests that higher capacity batteries require a longer duration to charge fully. For batteries with a power capacity ranging from 20 to 30 kWh, the average charging duration is at its minimum, approximately around 130 min. Conversely, the highest average charging durations are observed for batteries in the 71–80 kWh and the over 90 kWh categories, both nearing 440 min, or roughly 7.3 h. This correlation signifies the trade-off involved in electric vehicle design and operation. While larger battery capacities provide longer driving ranges and potentially less frequent charging needs, they also necessitate longer charging durations. This factor is important to consider in the context of ride-hailing services, where minimizing downtime (including charging time) is critical to maintain operational efficiency.

**Fig. 3d:**
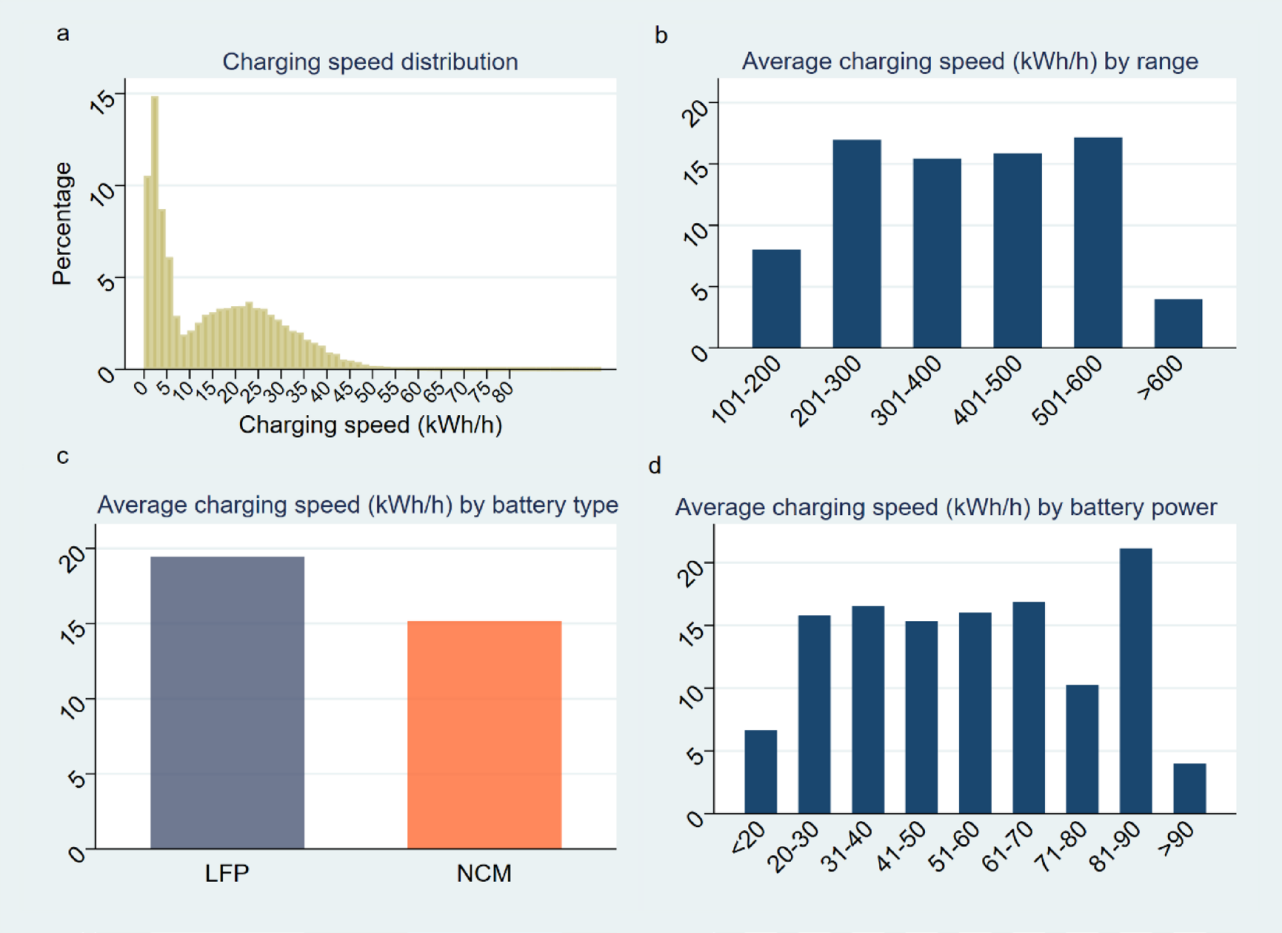
Charging Speed | **a**, Charging speed distribution.Two prominent peaks in charging speeds are observed—one at 2 kWh/h (15% of events) and another at 20–25 kWh/h (20% of events), indicating a mix of slower overnight charging and faster daytime charging.**b**, Charging speed by driving range: The average charging speed for most vehicles (200–600 km range) remains consistent at 15–17 kWh/h, regardless of the vehicle’s driving range, suggesting similar charging infrastructure needs for these vehicles.**c**, Charging speed by battery type: LFP batteries charge at an average speed of 20 kWh/h, faster than NCM batteries, which charge at 15 kWh/h. This difference is linked to the superior thermal stability and charge efficiency of LFP batteries.**d**, Charging speed by battery capacity: Charging speeds for most 20–70 kWh batteries are consistent at 15–17 kWh/h, indicating that factors other than battery capacity, such as battery chemistry and charger characteristics, influence charging speed. .

We proceed to report distribution of current charging speed of the electric vehicle ride-hailing fleet. Figure 3a portrays the distribution of charging speeds for the electric vehicle ride-hailing fleet. Two prominent peaks are noticeable in the distribution, indicating the most common charging speeds at which the EVs operate. The first significant peak is located at approximately 2 kWh/h, signifying that many charging events take place at this relatively slow speed. Roughly 15% of all charging events occur at this rate, which may correspond to slower, overnight charging practices or instances where only partial charging is required. The second notable peak is observed in the range of 20–25 kWh/h. This higher speed charging constitutes around 20% of all charging events, likely corresponding to fast-charging stations or instances where a quick charge is necessary during the day to maintain operations. Understanding the distribution of charging speeds provides insights into the charging infrastructure and practices utilized by the ride-hailing fleet. It can also inform strategies for optimizing charging behaviors and infrastructure upgrades to improve operational efficiency.

Figure 3b portrays the relationship between the average charging speed and the driving range of electric vehicles. According to the figure, the correlation between the average charging speed and the vehicle’s driving range is somewhat unclear. Most electric vehicles fall within the range of 200–600 km, as indicated by the central four bars on the graph. Interestingly, despite the variations in driving range, these vehicles exhibit very similar average charging speeds, primarily lying between 15 and 17 kWh/h. This observation implies that the charging speed does not scale proportionally with the vehicle’s driving range; instead, it seems to remain fairly consistent for most vehicles within this driving range bracket. The insight could have implications for fleet management and charging infrastructure planning, as it indicates that a broad range of EV models with varying driving ranges may be able to utilize similar charging infrastructure and maintain similar charging schedules.

Figures 2a and b and 3a, and 3b together offer a more complete view of how charging time and charging speed are influenced by driving range and other factors. Since charging time is determined by both charging speed and the amount of charge needed, check the interplay between these two variables is crucial for a more comprehensive understanding. Figure 2b, which shows the average charging duration by driving range, highlights that vehicles with a driving range of over 600 km require the longest charging time, averaging 430 min (over 7 h). This increase in charging time aligns with the need to charge larger batteries to a higher SOC. However, the relationship between charging time and driving range is not entirely linear, suggesting that other factors—such as battery capacity, charging technology—also play a role in determining charging process duration. When we look at Fig. 3b, which presents the average charging speed by driving range, we see that, despite differences in vehicle’s driving range, the charging speed remains relatively consistent for vehicles in the 200–600 km range (around 15–17 kWh/h). This appears to indicate that charging speed does not directly scale with driving range, suggesting that charging speeds are more influenced by factors like charger type, battery efficiency, or charging infrastructure rather than vehicle’s driving range alone. Here, it becomes clear that, overall, while longer-range vehicles have larger batteries that require more time to charge, they also benefit from faster charging speeds, which help mitigate the longer charging durations. In contrast, shorter-range vehicles may charge slower, contributing to their longer charging times, even though their batteries are smaller. Additionally, Fig. 2a (charging duration distribution) and Fig. 3a (charging speed distribution) complement the analysis by showing that while most charging events are relatively short, some still require significant time. This is particularly true for vehicles that charge at slower speeds (such as around 2 kWh/h), accounting for 15% of all events, which likely represent overnight charging or partial charging. Conversely, 20% of events occur at higher speeds (around 20–25 kWh/h), which likely correspond to fast-charging stations or situations where a quick recharge is needed to maintain fleet operations. The charging time-speed relationship is influenced by various factors. Vehicles with larger ranges tend to charge slower but benefit from faster charging speeds, whereas smaller vehicles with shorter ranges have longer charging durations due to slower speeds. Thus, optimizing charging infrastructure and understanding the charging behaviors associated with different vehicle categories can improve operational efficiency for the fleet.

Figure 3c reveals an interesting pattern concerning the influence of battery type on charging speed. Vehicles equipped with LFP batteries exhibit an average charging speed close to 20 kWh/h, which is noticeably higher than the average charging speed of NCM batteries, which is just above 15 kWh/h.

This result, when taken together with earlier observations regarding “Average charging initiation SOC by battery type” and “Average charging duration (Minutes) by battery type”, suggests a clear pattern. It appears that electric vehicles with LFP batteries generally have a higher average starting SOC, charge at faster speeds, and require shorter durations to charge fully when compared to vehicles with NCM batteries.

The reasons for these differences could be attributed to the inherent properties of the two battery types. LFP batteries are known for their excellent thermal stability, high charge and discharge efficiency, and long cycle life, which may contribute to their superior performance in these aspects. The implications of these findings are significant for electric vehicle users, fleet managers, and charging infrastructure planners. For instance, knowing that LFP batteries charge faster and require less time to reach a higher SOC may influence decisions on vehicle procurement, fleet management strategies, and the planning and utilization of charging infrastructure. This insight could also be used to optimize charging schedules and increase the efficiency of fleet operations.

Figure 3. presents the relationship between average charging speed and battery capacity. As per the data presented, the majority (99.24%) of battery capacities recorded lie within the range of 20–70 kWh. For these capacities, the average charging speeds appear to be similar, predominantly within the 15–17 kWh/h range.

Several factors could contribute to this lack of clear correlation. The charging speed of a battery does not rely solely on its capacity but is influenced by other aspects such as the battery’s internal resistance, the temperature, the charger’s power output, the battery management system, and the state of the battery (for instance, a nearly full or nearly empty battery will charge slower due to inherent battery chemistry constraints). These complexities make it difficult to draw a straightforward relationship between battery capacity and charging speed. However, knowing the typical charging speeds within the 20–70 kWh range might still provide useful information for electric vehicle users, service providers, and policy makers. For instance, it could be useful for planning the charging infrastructure, as it gives an indication of the average time an electric vehicle would occupy a charging station. Similarly, it might be relevant for users planning their trips, as it gives them an idea of the time they’d need to reserve for charging.

**Fig. 4:**
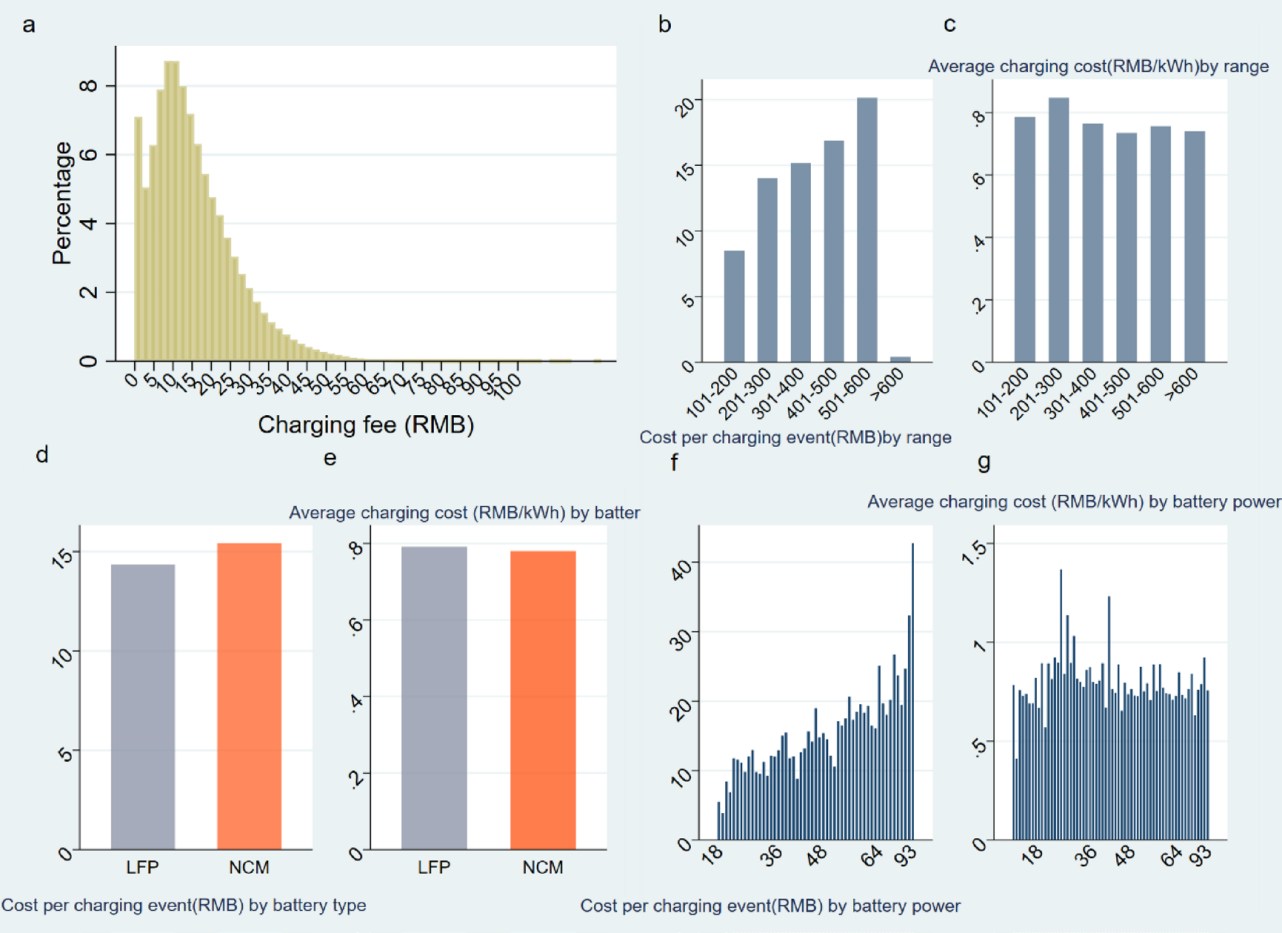
Charging Cost | **a**, Distribution of charging costs, with 60% of events costing less than 15 RMB, and the peak in the 8–12 RMB range reflecting common charging costs influenced by factors like electricity rates, energy required, and charging duration. **b**, charging cost by driving range, showing an average cost of 8 RMB for 100–200 km vehicles and 20 RMB for 501–600 km vehicles. **c**, Charging cost per kWh by driving range, with average costs ranging from 0.7 to 0.8 RMB across all vehicle ranges. **d**, Total cost per charging event for LFP and NCM batteries, with LFP batteries costing slightly less (around 14 RMB). **e**, Charging cost per kWh by battery type, with NCM batteries showing a slightly lower cost per kWh. **f**, Total cost per charging event by battery capacity, indicating a positive correlation with capacity, from 5 RMB for 18 kWh to 43 RMB for 93 kWh. **g**, charging cost per kWh by battery capacity, with costs remaining consistent between 0.6 and 0.8 RMB per kWh, regardless of capacity.

We proceed to report distribution of current charging cost of the electric vehicle ride-hailing fleet. Figure 4a shows that the majority of charging costs for the electric vehicle ride-hailing fleet are relatively low, with 60% of all charging events costing less than 15 RMB (Renminbi, the official currency of the People’s Republic of China). This finding is consistent with what we might expect, given that electricity costs tend to be lower than gasoline costs, one of the main selling points for electric vehicles.

The peak of the distribution within the 8–12 RMB range indicates that this is the most common cost for charging events. This could be due to a combination of factors including the prevailing electricity rates during the most common charging times, the average amount of energy required to charge the vehicles (which would be influenced by factors like the average SOC at the beginning of charging, and the efficiency of the vehicles), and the average length of charging sessions. The information gleaned from this graph could be quite useful for ride-hailing drivers when considering the potential operating costs of an electric vehicle. It could also be useful for policymakers and utilities when considering potential demand-response strategies or variable pricing schemes to manage electricity demand.

Figure 4b present the cost per charging event by driving range, which shows that BEVs with a range within 100–200 km incur an average charging cost of approximately 8 RMB, while those boasting a range of 501–600 km face an average cost of about 20 RMB. Figure 4c presents the charging cost per kWh by driving range, indicating for all ranged BEVs, the average charging cost per kWh is between 0.7 and 0.8 RMB.

Figure 4d and e offer a valuable comparison between two different battery types (LFP and NCM) in terms of average charging cost. Figure 4d shows the average total cost per charging event for both LFP and NCM batteries. Here, we see that the total cost for LFP batteries is slightly less, on average, than for NCM batteries by about 1 RMB, averaging around 14 RMB per charging event. Figure 4e, however, depicts the average charging cost per kWh with respect to driving range. Interestingly, this graph reveals that the per kWh cost is slightly lower for NCM batteries.

**Fig.** 4f and 4 g present charging cost by battery capacity. As depicted in the Fig. Figure 4f, the average total cost per charging event displays a positive correlation with battery capacity, extending from 5 RMB for 18 kWh batteries to approximately 43 RMB for 93 kWh batteries. Figure 4 g, however, offers a different perspective by showing the average cost per kWh for batteries of different capacities, indicating that the cost per kWh remains relatively stable, with the majority of BEV charging cost falling within a narrow range of 0.6–0.8 RMB per kWh. This suggests that while larger-capacity batteries might require a higher total cost to fully charge, they do not necessarily become more expensive on a per-kWh basis.

Furthermore, to optimize the fleet’s overall charging efficiency and minimize both charging costs and carbon emissions, predictive models can be developed based on the factors we’ve explored above. By incorporating variables like SOC at the start, battery type, charging speed, and vehicle range, we can apply a neural network model to predict the lowest charging costs and carbon emissions for each event. The neural network can learn complex relationships between these variables and provide optimal charging strategies, helping fleet operators choose the best charging times and facilities to reduce costs while maintaining operational efficiency. This predictive approach is crucial in achieving sustainable fleet management in electric vehicle ride-hailing services.

## Predicted charging cost

**Fig. 5:**
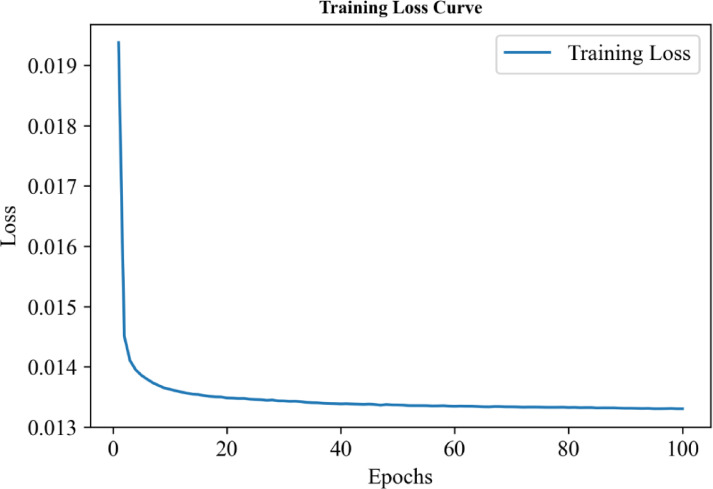
The variation of the training loss over 0 to 100 epochs.

We turn to report the predicted charging cost based on optimization using Adam algorithm and neural network for the electric vehicle ride-hailing fleet. Figure 5 reports the training loss curve, indicating that after training the model for 100 epochs with 10 iterations, the training loss gradually decreased, converging around the 20th epoch. On the test set, our model achieved a loss rate of 1.23%. The experimental results demonstrate that the Adam optimizer performed well on the training dataset.

**Fig. 6:**
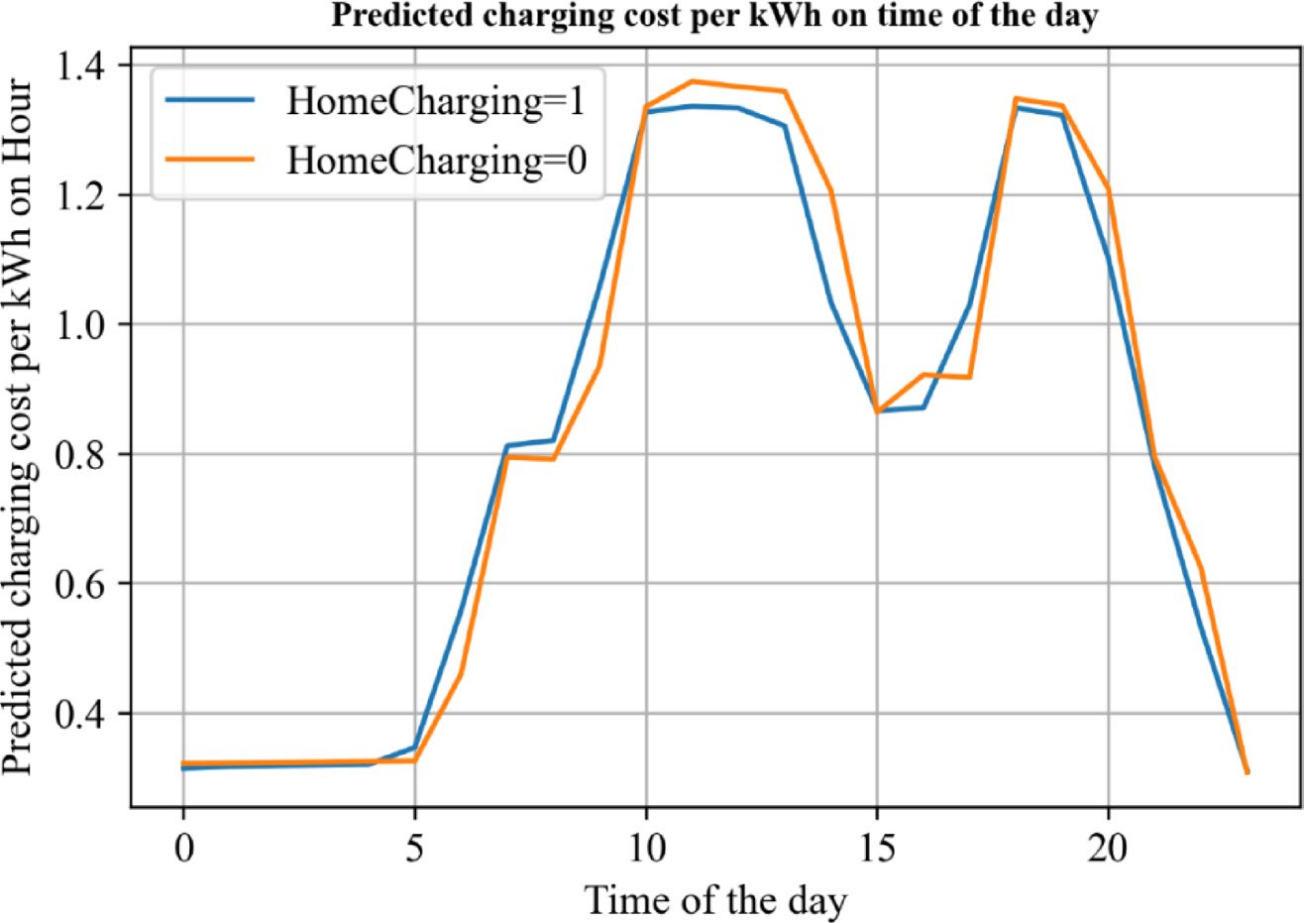
Predicted charging cost per kWh on time of the day.

Using the trained deep learning model, we made predictions for the charging expenses at different charging starting time. Figure 6 reports the predicted charging cost on time of the day. The unit electricity expense appears to be lowest between 0:00 and 5:00 in the early morning hours, making it the most cost-effective time for charging. The expense during this period is nearly a third of what it would be during peak hours. This may be due to lower overall power grid load during the night, which improves charging efficiency and subsequently reduces the cost.

Conversely, the highest costs for charging are observed from 10:00 AM to 2:00 PM and 6:00 PM to 7:00 PM. These time intervals correspond to peak electricity usage hours when the demand for electricity is high, resulting in increased costs. Interestingly, the neural network analysis indicates that charging outside typically incurs higher electricity expenses compared to home charging across most time intervals, likely due to service fees and infrastructure differences. The discrepancy in costs is particularly notable during the peak electricity usage periods. As demand for electricity continues to grow, the cost difference between home charging and outside charging is expected to widen further.

**Fig. 7:**
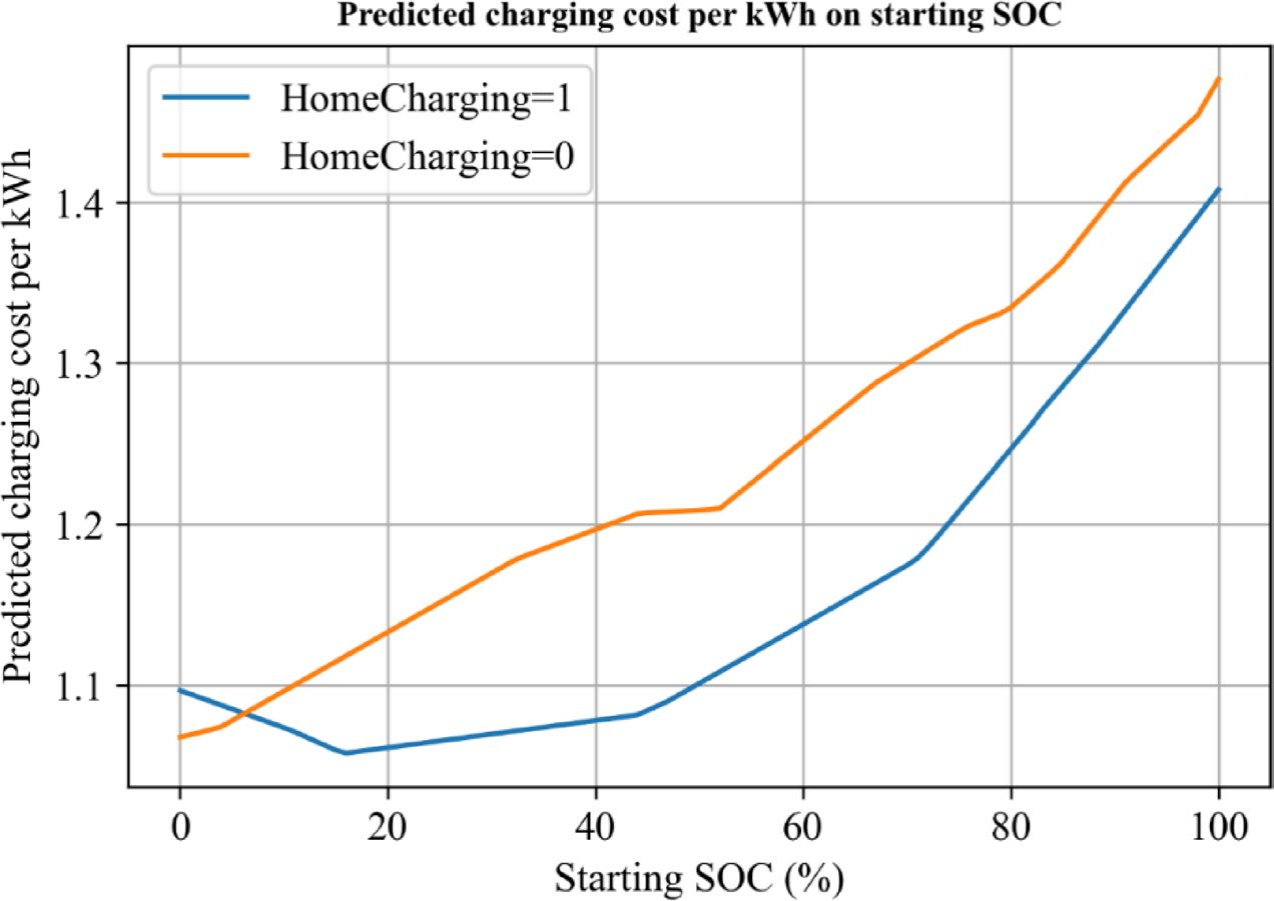
Predicted charging cost per kWh on time on starting SOC.

To further explore the optimal starting SOC for charging, we used the trained model to predict the unit electricity price for different starting SOC, as shown in Fig. 7. The results revealed that, overall, as the starting SOC increased, the unit electricity expenses for charging continued to rise.

However, when considering the scenario of charging at home, the starting SOC at around 18% reached the minimum value for the unit electricity expenses. After the starting SOC exceeded 80, the difference in unit electricity expenses between the two charging scenarios gradually decreased. Considering that the majority of starting SOC values in the sample population are concentrated between 20% and 60%, which is the primary reason for higher vehicle usage costs, changing charging habits could significantly reduce their overall vehicle usage costs.

In summary, based on the model predictions, it is evident that the starting SOC plays a crucial role in determining the unit electricity expenses for charging an electric vehicle. For charging at home, the optimal starting SOC is around 18%, which results in the lowest electricity expenses. However, for starting SOC values above 80, the differences in unit electricity expenses between charging scenarios diminish. Therefore, encouraging users to adopt charging habits with lower starting SOC values could substantially reduce their overall vehicle usage costs.

**Fig. 8:**
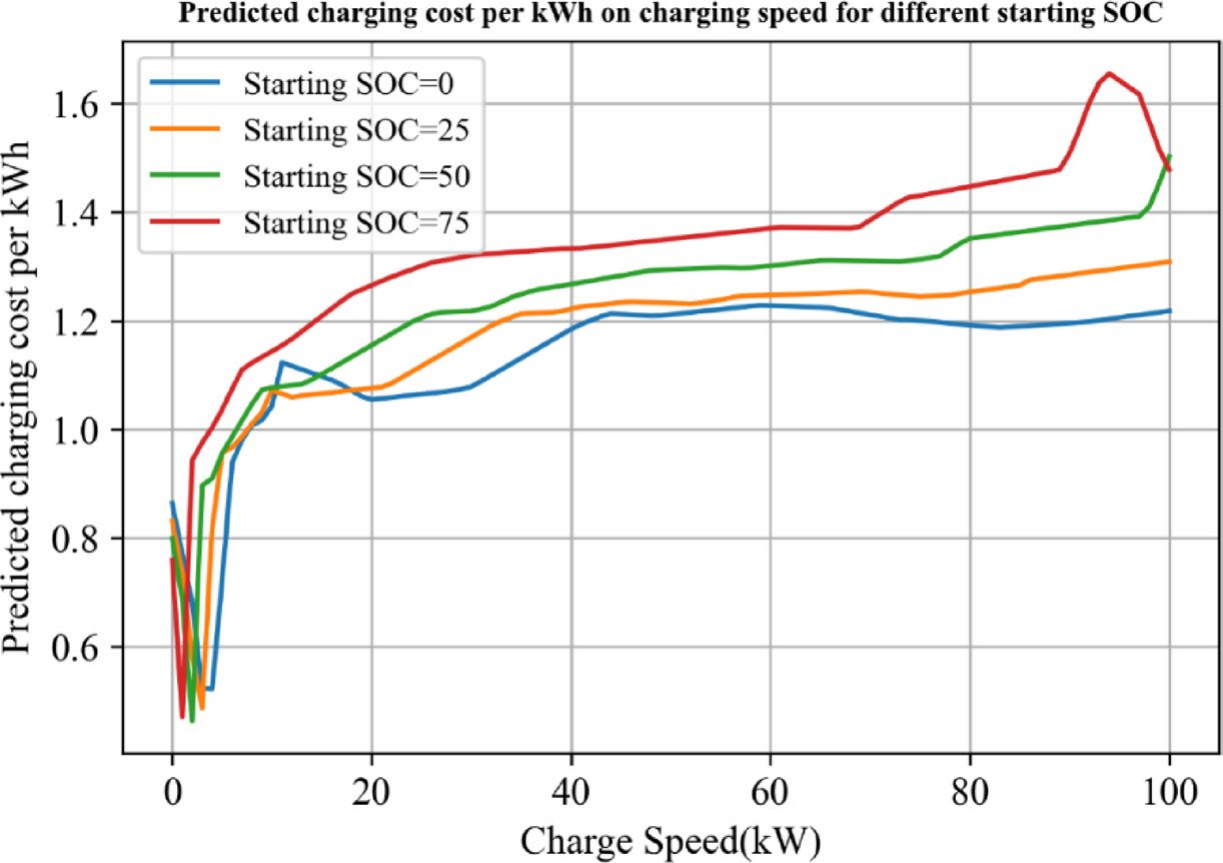
Predicted charging cost per kWh on charging speed for different starting SOC.

Figure 8 provides an insightful analysis into the relationship between the optimized charging cost, charging speed, and the starting SOC. The findings suggest that as the starting SOC decreases, the cost of electricity per kilowatt-hour also decreases. This indicates that initiating charging when the SOC is low can help users save on their charging cost. However, as charging speeds increase, the cost benefits associated with lower starting SOC strategies seem to diminish. Particularly, when the charging rate ranges from 0 to 20 kW, the unit electricity expenses escalate rapidly. Beyond 20 kW, though, the rate of increase decelerates.

Interestingly, under high charging rates, the unit electricity expenses for high starting SOC decrease. This observation suggests that as charging infrastructure improves and allows for higher charging rates, the cost benefits of low starting SOC charging strategies may eventually be superseded by high starting SOC strategies.

## Predicted carbon emissions

**Fig. 9:**
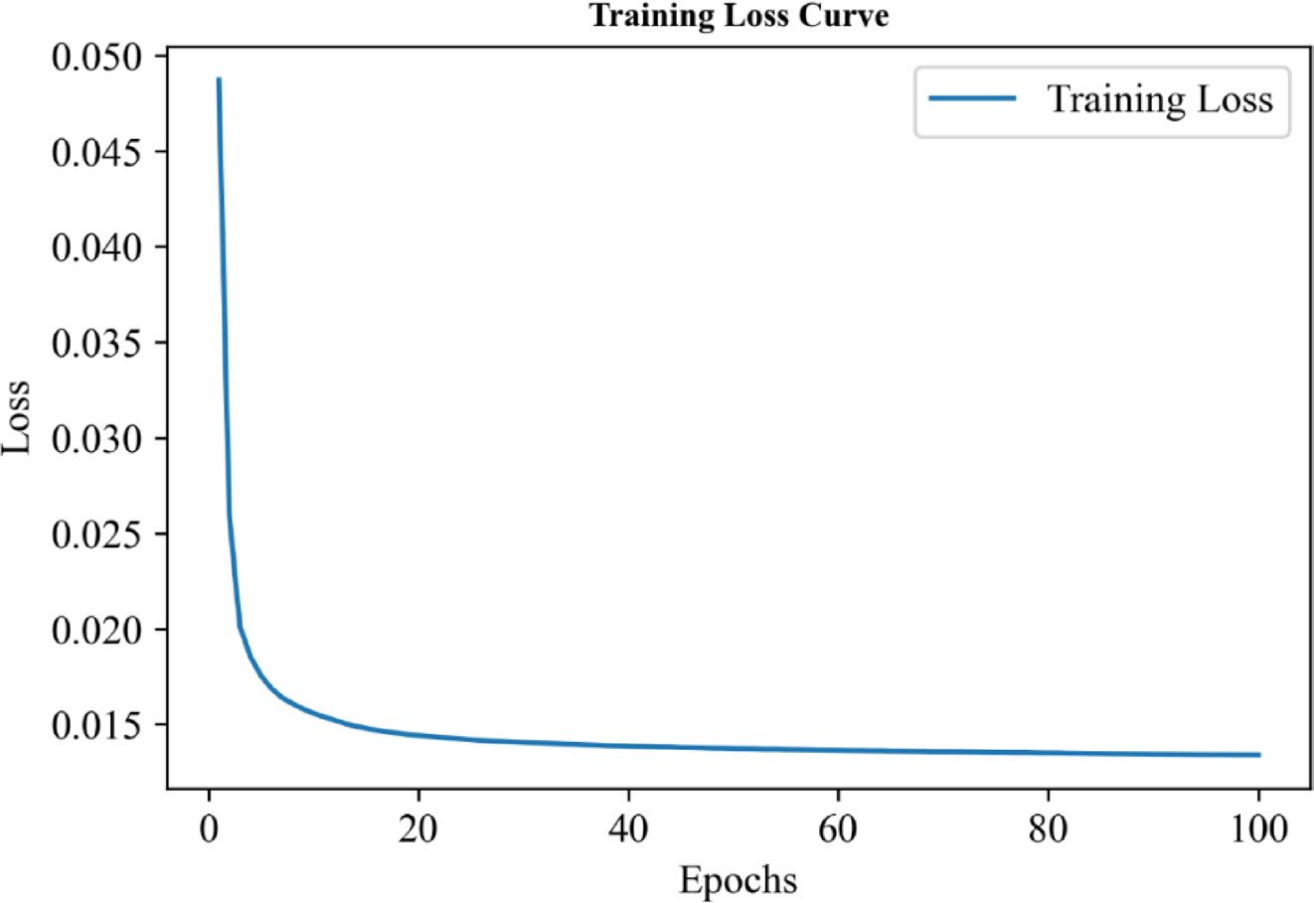
The variation of the training loss over 0 to 100 epochs.

We turn to report the predicted carbon emissions based on optimization using Adam algorithm and neural network for the electric vehicle ride-hailing fleet. Figure 9 reports the training loss curve, indicating that after training the model for 100 epochs with 10 iterations, the training loss gradually decreased, from an initial 5% to approximately 1.5%. Remarkably, the final test set sample loss achieved an impressive value of around 1.3%. These results unequivocally indicate that the model training exhibited remarkable proficiency and efficacy.

**Fig. 10:**
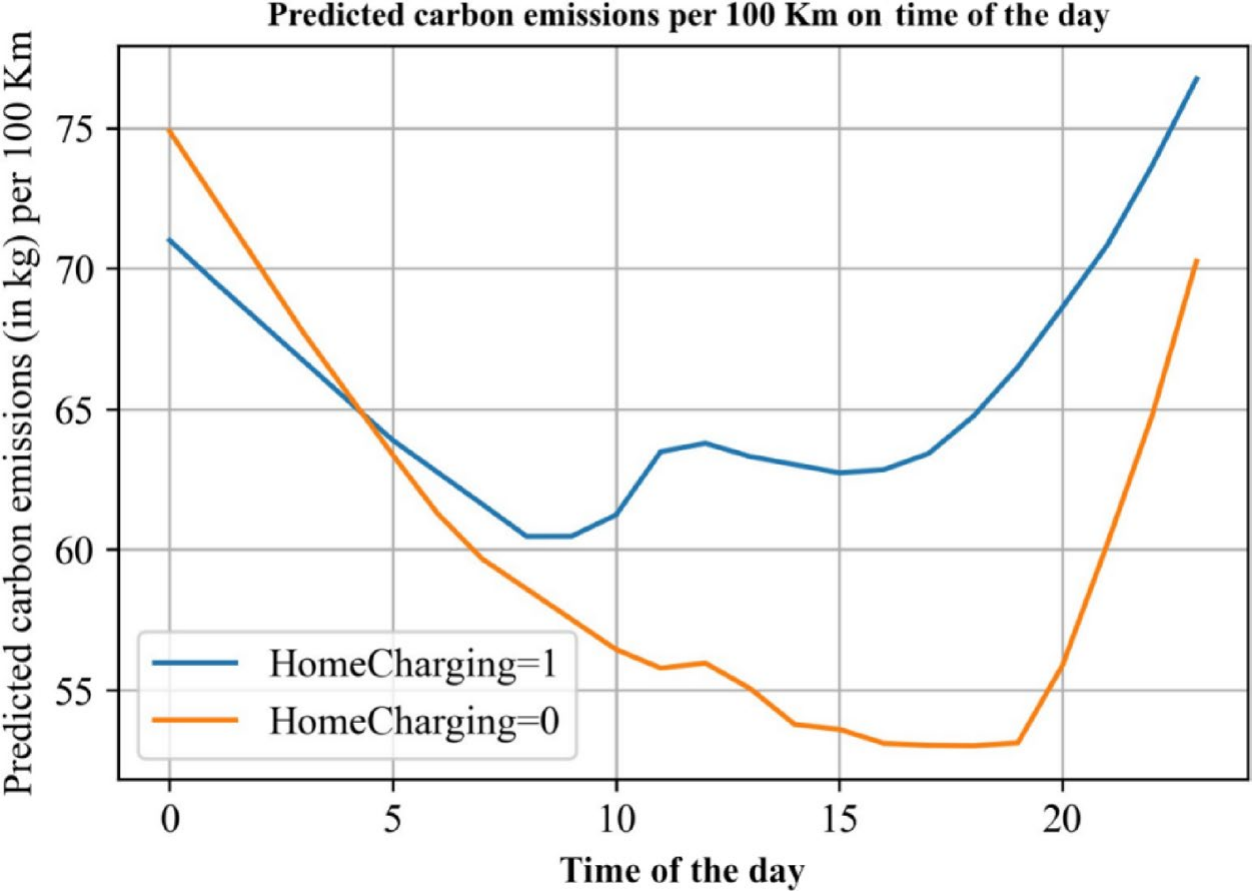
Predicted carbon emissions per 100 km on time of the day.

Figure 10 presents the model’s predictions regarding the carbon emissions per 100 km based on the time of the day. The results reveal a fascinating pattern between home and outside charging. During daytime hours, charging electric vehicles at home results in higher CO2 emissions per hundred kilometers compared to charging outside. In contrast, during the nighttime period from midnight to 5:00 AM, home charging leads to lesser CO2 emissions than outside charging.

For home charging, the lowest points of CO2 emissions per hundred kilometers occur around 9:00 AM and 4:00 PM. This could be attributed to the fluctuations in the grid’s carbon intensity, possibly due to the contribution of renewable energy sources during these times. In contrast, for outside charging, the least CO2 emissions per hundred kilometers are observed around 7:00 PM.

**Fig. 11:**
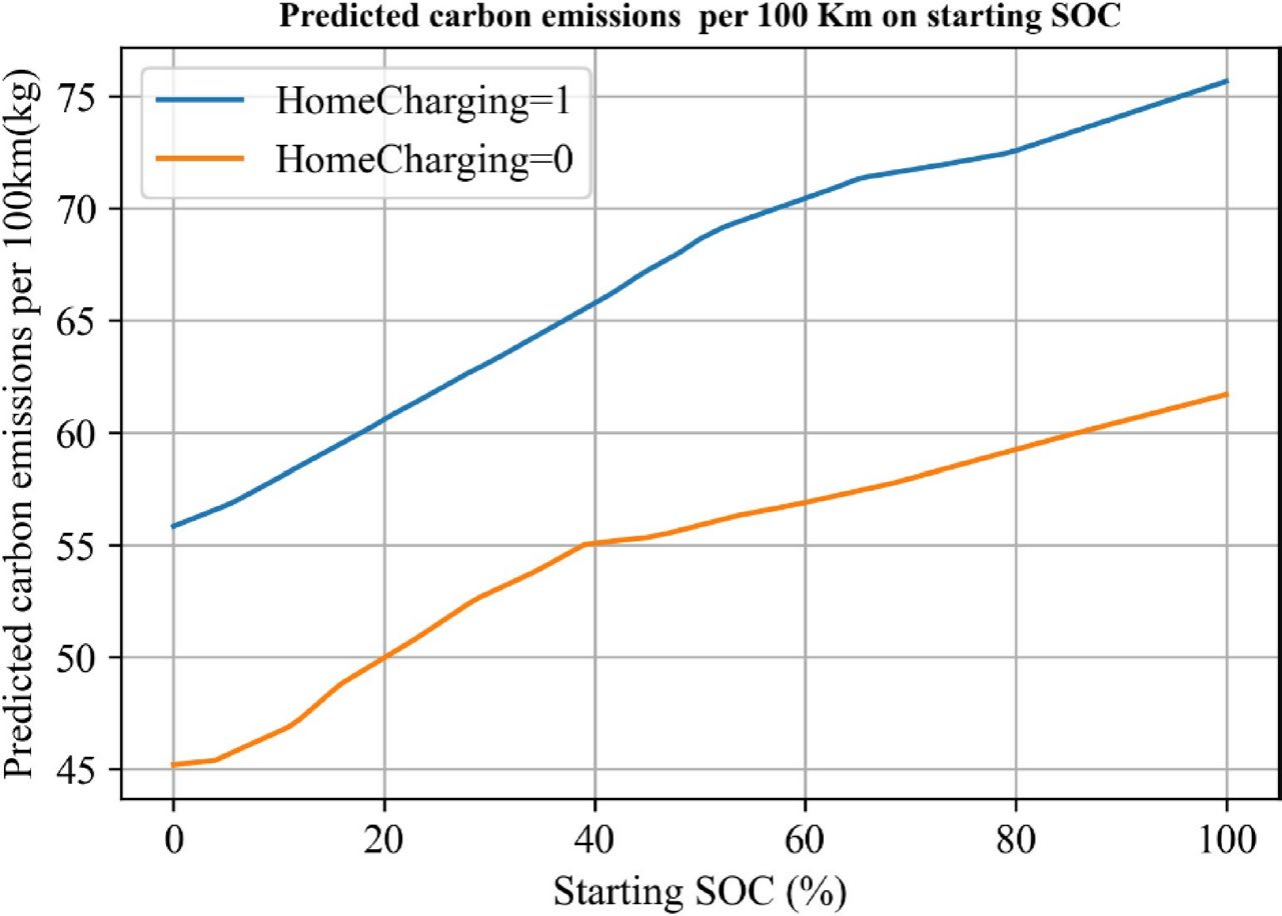
Predicted carbon emissions per 100 km on starting SOC.

Figure 11 presents the model’s predictions regarding the carbon emissions per 100 km based on the starting SOC. The figure presents an interesting, relatively steady correlation between starting SOC and carbon emissions. As the starting SOC decreases, the CO2 emissions per hundred kilometers also gradually decrease. This suggests that initiating charging when the battery’s SOC is low will result in lower carbon emissions, making it a more environmentally friendly approach. On the contrary, charging when the battery’s SOC is high can lead to approximately 50% more CO2 emissions per hundred kilometers compared to starting from 0 SOC.

An important note is that, regardless of the starting SOC scenario, charging outside consistently results in lower carbon emissions compared to charging at home. This might be due to more efficient grid utilization and potentially greener power sources at public charging stations.

**Fig. 12:**
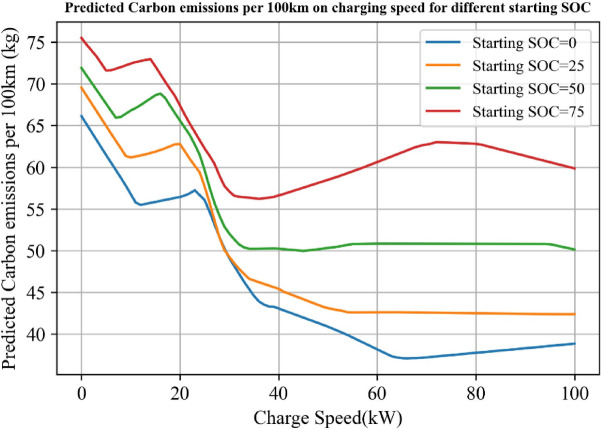
Predicted carbon emissions per 100 km on charging speed for different starting SOC.

Figure 12 presents the model’s predictions regarding the carbon emissions per 100 km based on charging speed for different SOC. The findings show an intriguing correlation between charging speed and carbon emissions. As charging speed increases, especially within the range of 0 to 40 kW, carbon emissions per hundred kilometers decrease rapidly, stabilizing after the charging speed reaches 40 kW. This indicates that high charging speeds, often associated with public charging stations, are a primary factor in the observed difference in CO2 emissions per hundred kilometers between home and outside charging.

Another observation reiterated by this model is that lower starting SOCs lead to reduced CO2 emissions per hundred kilometers. This means that a combination of charging at a low SOC and high charging speeds result in the lowest carbon emissions. Therefore, charging outside, which often involves fast-charging stations, when the battery’s SOC is low, appears to be the most eco-friendly strategy. These insights could be essential in guiding user behavior and forming policies to minimize the environmental impact of electric vehicle usage. They underline the environmental advantages of charging outside at fast-charging stations, especially when the battery’s SOC is low.

## Discussion

This study offers an extensive analysis of electric EV charging behaviors among ride-hailing drivers in Beijing, based on a large dataset comprising 2.14 million charging events from 9,221 unique EVs in the ride-hailing fleet. The key metrics evaluated in this study include initial SOC, average charging duration, charging speed, and average cost per charging event.

First, regarding the initial SOC, our analysis shows that ride-hailing operators predominantly initiate charging when the SOC ranges from 20 to 60%. Merely 2% of charging events are started when the SOC is less than 5% or more than 90%. Interestingly, LFP batteries commence charging at an average SOC of 57%, while NCM batteries start at a considerably lower average SOC of 41%. This highlights significant disparities between the two battery types. The dataset also suggests that larger battery capacities are associated with a higher average initial SOC. Second, pertaining to the average charging duration, our findings indicate that 60% of charging events are concluded within 2 h, with around 18% of all charging events occurring around the one-hour mark. No definitive correlation between the average charging duration and vehicle range is discerned, although data suggests that vehicles across most range categories have an average charging duration around the 5-hour mark. LFP batteries show a significantly shorter average charging duration (140 min) compared to NCM batteries (240 min). The dataset shows a 10:1 ratio of NCM to LFP charging events, suggesting that the observed differences in charging duration may be influenced by the higher number of NCM batteries in use. Meanwhile, larger battery capacities generally corresponded with longer charging duration. All battery capacities had an average charging duration of between 2 and 8 h, with the shortest being the 20-30kWh range at about 130 min, and the longest being the 71-80kWh and above 90kWh ranges at around 440 min. Third, with respect to the average charging speed, our findings indicate that 15% of all charging events operate at around 2kWh/h, falling under the category of slow charging. About a third of all charging events have charging speeds less than 5kWh/h, and nearly a fifth of all charging events occur within the range of 20-25kWh/h. The relationship between average charging speed and vehicle range remains ambiguous, but most vehicles in the 200–600 km range have an average charging speed of around 15-17kWh/h. LFP EVs have an average charging speed of around 20kWh/h, outpacing NCM vehicles, which slightly exceeded 15kWh/h. No clear association is identified between battery capacity and average charging speed. Fourthly, as it pertains to the average charging cost, our study revealed that 60% of charging costs do not exceed 15 RMB, with around 17% of events incurring costs within the 8–12 RMB range. Most charging costs do not exceed 60 RMB. A general positive correlation exists between vehicle range and average cost per charging event. In terms of battery type, LFP batteries have a lower average total charging cost per event (around 14 RMB), but NCM batteries are less expensive in terms of the average cost per kWh. For battery capacity, the total average cost per charging event generally correlates positively with battery capacity, ranging from about 5 RMB for 18kWh batteries to approximately 43 RMB for 93kWh batteries. However, the average cost per kWh shows no distinct relationship with battery capacity, with most values falling within the 0.6–0.8 RMB range.

Overall, these findings provide a rich and nuanced understanding of charging behaviors among ride-hailing drivers in Beijing. They offer valuable insights for prospective EV owners or operators (including ride-hailing drivers) and can inform decisions related to battery type selection, charging schedules, and cost management. The findings also have potential implications for infrastructure planning and policy-making, as they highlight the importance of considering the specific characteristics and charging requirements of different battery types in designing charging stations and developing regulations and incentives for EV use.

This study leverages deep learning neural network and the Adam optimization algorithm to optimize the charging behavior of electric vehicle ride-hailing fleet under the goal of minimizing charging cost and reducing carbon emissions. The specific charging strategies include initiating charging time, starting SOC, and charging location selection.

Specifically, our analysis reveals that the goal of minimizing costs and carbon emissions often conflict when choosing the initiating charging time. Nighttime charging significantly reduces charging cost but contributes to higher carbon emissions, particularly in non-home-based charging scenarios. The data suggests that charging around 15:00 provides a balanced optimization goal, both lowering charging costs while reducing carbon emissions.

Meanwhile, lowering the starting SOC is associated with a 12% reduction in charging costs and a 9% decrease in emissions per event, suggesting potential improvements if operators adjust their charging thresholds. However, the data shows that as electric vehicle ride-hailing operators often start charging within the 20–60% SOC range, primarily due to range anxiety and limited access to public charging stations. Our model therefore suggests that government should encourage electric vehicle ride-hailing operators to lower their starting SOC to enjoy the substantial benefit both in money and environment.

In terms of charging speed, our optimization model suggests that increasing the charging speed from 0 to 20 kW considerably raises the charging cost, while increasing it between 0 and 40 kW decreases carbon emissions. Therefore, we suggest that for practical purposes, maintaining charging speed between 20 kW and 40 kW might be a more effective and realistic optimization charging strategy for electric vehicle ride-hailing operators.

Finally, as expected, home charging proves to be the most cost-effective mode, with non-home-based charging costs representing a conservative estimate, excluding service fees and additional expenses. However, home charging often leads to higher carbon emissions due to the generally slower charging speed. Therefore, we suggest that future upgrades to home charging stations to speeds above 20 kW could help narrow the carbon emissions gap between home and outdoor charging.

The findings of this study underscore the importance of analyzing charging behaviors to identify actionable improvements for BEV ride-hailing services. By quantifying trade-offs between cost and emissions, we provide insights for operators to refine their strategies. These strategies can strike a balance between cost-effectiveness and carbon emissions reduction, paving the way for a greener and more economical transportation landscape. Policymakers and ride-hailing service providers can leverage these insights to create informed guidelines for efficient charging behaviors, and BEV ride-hailing drivers can use these insights to make more sustainable decisions. In this way, the BEV ride-hailing sector can play a significant role in fostering sustainable urban mobility.

AI-based optimization techniques play a significant role in ensuring the efficient and effective integration of EVs into the power grid, fostering the widespread adoption of EVs and the growth of sustainable energy systems. The economic repercussions of optimized EV ride-hailing fleet charging behavior can be analyzed from two perspectives: that of the power grid and that of EV ride-hailing operators. From the perspective of the power grid, the integration of additional electric vehicle load may require an expansion of power generation capacity, subsequently influencing generation costs and the investment in charging infrastructure. These factors in turn bear implications for the total cost of electric vehicle usage. Conversely, from the viewpoint of EV ride-hailing operators, costs encompass both the initial acquisition expense and the cost of usage, the latter being significantly influenced by their charging behavior. The analysis of charging patterns through AI models can inform tariff design and pricing policies, enabling stakeholders to balance operational efficiency with grid stability, thereby effectively curbing costs for both the power grid and EV ride-hailing operators. This mutual benefit can enhance the profitability for stakeholders. By utilizing these simulation-based, optimized intelligent charging strategies, stakeholders are empowered to make well-informed decisions regarding tariff coordination and pricing policies. This leads to cost efficiencies, promotes EV uptake, and ensures grid stability in line with sustainable development. Our study quantifies how variables such as peak-valley periods, initial SOC, and charging speed influence electricity costs, highlighting opportunities for operators to adjust behaviors under current infrastructure. Such findings are particularly beneficial for electric vehicle users, enabling them to strategically plan their charging schedules to minimize costs and carbon emissions. Charging at home during off-peak hours can significantly reduce electricity costs. Furthermore, these insights also impart valuable knowledge to both EV users and policymakers, emphasizing the importance of considering the SOC at the commencement of charging and the charging location in managing the carbon footprint of electric vehicles.

The environmental impact of EVs has engendered considerable debate. Proponents contend that EVs, devoid of tailpipe emissions, represent a more sustainable and environmentally friendly alternative. However, critics argue that a comprehensive lifecycle assessment of EVs, which includes their production, disposal, and the additional electricity generation required for charging, could still yield substantial greenhouse gas emissions. Nonetheless, the prevailing consensus supports the notion that EVs, compared to traditional internal combustion engine vehicles (ICEVs), confer a more positive influence on carbon emission reduction. This impact can be further augmented through optimized charging strategies, including the selection of eco-friendlier battery types, optimization of battery range, and curbing the average number of charging cycles. Our research extends to simulating and modeling carbon emissions per hundred kilometers using AI algorithms, thus providing a theoretical basis for stakeholders to reduce the carbon footprint of electric vehicles. These insights hold potential for guiding EV owners and policymakers in devising strategies to mitigate the carbon footprint associated with EV charging. For instance, incentives could be established for charging during periods of low carbon intensity, or infrastructure development could be prioritized to increase the proportion of renewable energy in the grid. Potential adverse impacts on the power grid predominantly encompass harmonic distortion, system losses, voltage drops, phase imbalances, elevated power demand, equipment overloads, and overall stability issues. The advent of Vehicle-to-Grid (V2G) technology offers promise in balancing load profiles and curtailing system losses. Our research findings robustly support demand response measures and aid in optimizing the location of charging infrastructure and charging rates.

Our study does have some limitations, in our analysis of the charging behavior, we observed a correlation between the initial SOC and the charging cost. However, it is important to note that charging costs are not solely influenced by the initial SOC, but also by the supply price from the grid, which can be affected by factors such as the time of day, seasonal variations, and regional differences. For example, charging during peak hours tends to be more expensive compared to off-peak hours. Additionally, seasonal factors such as temperature can influence electricity demand, thereby affecting grid prices. Therefore, while the initial SOC plays a role in determining the charging cost, the observed correlation may also reflect the influence of these external factors. It is critical to emphasize that our study focuses on analyzing current charging behaviors under static historical conditions, which limits our ability to account for dynamic feedback effects, such as changes in electricity prices or grid emissions due to widespread adoption of recommended strategies. Our findings suggest that charging during off-peak hours and in areas with lower electricity prices can significantly reduce the overall cost. To address these limitations and achieve true systematic optimization, future research should integrate dynamic feedback mechanisms, such as real-time grid pricing and renewable energy availability, into charging strategy models. By incorporating these variables, researchers can develop adaptive frameworks that not only minimize costs but also enhance grid stability and reduce carbon emissions, paving the way for a more sustainable and efficient EV ecosystem.

The research findings delineated in this study suggest several avenues for further research. Firstly, this study, anchored in representative driving and charging data from Beijing’s ride-hailing EVs, could be broadened to encompass additional regions and diverse types of EVs, enriching the global understanding of EV usage. Secondly, the present study’s methodological approach employing a deep learning neural network with the Adam optimization model for simulation could be expanded. Future research could incorporate a range of a AI algorithms, such as genetic algorithms (GA) or particle swarm optimization (PSO), further enhancing the optimization process. Thirdly, while the current study honed in on optimizing charging strategies based on minimizing the unit charging cost and carbon emissions, future investigations could widen the optimization objectives. This could include strategies aimed at alleviating stress on the power grid or employing route-based charging strategies, among others. Lastly, the methodology adopted in this study of matching the latitude and longitude data of residential areas in Beijing with electric ride-hailing charging locations, segregated into home charging and out-of-home charging, could be further refined. Future research endeavors could encompass a more granular delineation of charging scenarios, including public charging, workplace charging, and fast charging. These suggested trajectories hold promise for contributing to a more robust and diverse understanding of EV charging scenarios, potentially facilitating the evolution of advanced and efficient charging strategies within the forthcoming EV ecosystem.

## Methods

### Dataset of driving and charging events for electric vehicle ride-hailing fleet

This research capitalizes primarily on two comprehensive data resources. The inaugural dataset emanates from the National Monitoring and Management Platform for New Energy Vehicles(http://www.ndanev.com). This platform, an official large-scale data repository, has received endorsement from China’s Ministry of Industry and Information Technology. The investigation specifically hones in on actual driving and charging occurrences pertaining to battery electric vehicle ride-hailing fleets operating within the boundaries of Beijing. The period under scrutiny spans from January 2019 to October 2020.

To guarantee the representativeness of the data, we operated several steps to clean the data Initially, charging events where no discernable alterations in the SOC were observed before and after the charging phase were eliminated. Additionally, charging instances where the initial SOC exceeded 99% were dismissed to ensure that each observation represented a legitimate charging event. Subsequently, charging events were winsorized based on a 99% threshold related to the charging duration, charging speed, battery power, and vehicle range. Upon the application of these stringent data purification parameters, approximately 2.14 million charging events related to 9,113 ride-hailing battery electric vehicles (BEVs) r *RMB* emained. Driving events were similarly winsorized based on a 99% threshold, considering parameters such as driving speed, battery power, and vehicle range. This procedure yielded approximately 10.71 million driving events, involving 10,067 vehicles. Figure 1 illustrates the count of ride-hailing BEVs by month, indicating a pervasive ascending trend from January 2019 to December 2019. This upward trajectory was observed both for BEVs involved in charging and driving events. However, the count of BEVs exhibited considerable fluctuation throughout 2020, likely attributable to the impact of the COVID-19 pandemic. Particularly, the most drastic reductions in BEV counts were witnessed in February 2020 and October 2020.

## Dataset of Geospatial digital map

The second data repository leveraged in this study stems from the 2018 Geospatial Digital Map of Beijing, generously furnished by Gaode Map, one of the most comprehensive digital map providers in China. This dataset is intrinsically vector-based, amenable to manipulation, and adheres to the World Geodetic System 1984 Web-Mercator projection standard. The dataset incorporates more than half a million data entries, partitioned into 14 distinct categories of Points of Interest (POIs). These categories encapsulate a diverse array of domains including, but not limited to, catering services, commercial and residential establishments, significant landmarks, and public infrastructure. Each specific category comprises data attributes such as the POI’s name, geospatial coordinates (longitude and latitude), and its corresponding classification.

From this abundant dataset, we engaged in selective filtration to extract entries pertinent to residential domains. This process yielded a subset characterized by geographical coordinate information linked to a total of 11,214 residential communities.

## Featuring charging events based on the K-nearest neighbors algorithm

Although clustering techniques like K-means are commonly used in spatial data analysis, we chose the K-Nearest Neighbors (KNN) algorithm for classifying charging events based on geographic proximity to residential areas. The primary motivation for this decision was the need to distinguish between home-based and non-home-based charging events, which was essential for understanding the spatial distribution of charging behaviors. We used the Ball Tree algorithm to implement KNN, where charging events were classified as ‘home charging’ if the charging location was within 500 m of a residential community. This method was particularly suitable because it enabled us to incorporate precise geographic coordinates and apply the Haversine formula to calculate distances between charging locations and residential communities. We relied on the 11,214 residential communities delineated in the geospatial digital map dataset, which encapsulates precise geographical coordinates (longitude and latitude) of myriad residential communities throughout Beijing.

The distance between two points ($\:{lng}_{1}$, $\:{lat}_{1}$) and ($\:{lng}_{2}$, $\:{lat}_{2}$) on the Earth’s surface is calculated using the haversine formula:

1 $$\:a={sin}^{2}\left(\frac{\varDelta\:lat}{2}\right)+\text{cos}({lat}_{c})\times\:cos({lat}_{r})\times\:{sin}^{2}(\frac{\varDelta\:lng}{2})\:$$

2 $$\:d=2\times\:a\text{sin}\left(\sqrt{a}\right)\times\:R$$.

Where:

### $\:\varDelta\:lat$

The difference in latitude between the two points.

### $\:\varDelta\:lng$

The difference in longitude between the two points.

### $\:{lat}_{c}$

The latitude of the charging station.

### $\:{lat}_{r}$

The latitude of the residential community.

### $\:R$

The radius of the Earth. Here, it is set to be 6371 km.

### $\:a$

An intermediate variable calculated based on Eq. (1).

### $\:d$

The haversine distance between the two points. It is calculated as d = $\:2\times\:a\text{sin}\left(\sqrt{a}\right)\times\:$ 6371 $\:\times\:$ 1000.

Leveraging the aforementioned Haversine formula, the great-circle distance between any given pair of geographic coordinates was computed. Subsequently, a KNN classifier was designed, predicated on the Ball Tree algorithm. Charging events were classified as ‘home charging’ if the charging location was situated within a radius of 500 m from the nearest residential community. In contrast, all other charging events were designated as ‘non-home-based charging’.

Taking into account that some residential communities in Beijing are relatively small and the city has a charging pile coverage rate higher than the national average, respectively present the daily time distribution of home vs. non-home-based charging events when defining home charging as within 250 m, 125 m, and 62.5 m, respectively (see in Supplementary Information Fig. S.1., Fig. S.2.and Fig. S.3.). Both categories, home charging and non-home charging, exhibit a conspicuous trough during the interval of 2 am to 10 am. The zenith for home charging is attained at 4 pm, while non-home charging achieves its climax at 11 pm. These observations resonate profoundly with the findings disseminated in the 2019 Beijing New Energy Vehicle Charging Behavior Report, published by the Beijing Public Charging Infrastructure Data Information Service Platform. This report identifies 11 pm as the apex of charging activity within the city.

### Estimating charging cost by matching charging events with real-time grid power price

Following this, we proceeded to calculate the charging cost for each charging event from the perspective of the consumer. Given that all EV charging service providers in Beijing adopt a Time-of-Use (TOU) tariff structure—where pricing fluctuates based on the time of energy consumption—we incorporated the following steps to process the pertinent variables within our primary dataset:

Step I: We initially divided the 24-hour periods spanning from January 2019 to October 2020 into four classifications, congruent with Beijing’s official real-time grid power pricing scheme: extreme-peak periods, peak periods, flat periods, and valley periods. The precise segments were delineated as follows: peak periods (10:00–15:00, 18:00–21:00), flat periods (7:00–10:00, 15:00–18:00, 21:00–23:00), valley periods (23:00–7:00), and summer extreme-peak periods (July to August: 11:00–13:00, 16:00–17:00).

Step II: Drawing upon official documents issued by the Beijing municipal power authority, we ascertained the electricity price (RMB/kWh) for each temporal segment from January 2019 to October 2020. Owing to an absence of data concerning the charging piles utilized in the charging events, and the fact that charging piles in numerous Beijing communities may adhere to both residential and commercial power pricing standards due to local determinants, we initially selected the pricing standard for voltage levels below 1 kV under the category of general urban commercial power. This pricing standard might lead to an approximate overestimation of RMB 0.04 per kWh.

Step III: Each charging event was then categorized into the precise segments described in step I, according to the starting and ending time of each charging event. The total energy consumption and charging cost for each charging event was subsequently calculated by multiplying the real-time grid power price with the corresponding duration of charging events in each segment.

Concerning non-home-based charging events, service fees are typically imposed in addition to the cost of electricity. For example, on July 30, 2023, the Teld EV charging station at Beijing Times Square charged a service fee of RMB 0.8 per kWh (Charging station on Gaode map link: https://www.amap.com/place/B0JRRAQB3I). However, different charging service providers and distinct charging stations (e.g., State Grid, Teld, StarCharging, etc.) adopt varying service fee standards. On July 30, 2023, both the State Grid EV charging station and the Teld EV charging station levied a service fee of RMB 0.8 per kWh throughout the day, while StarCharging charged merely RMB 0.3–0.4 per kWh, and in some instances, service fee are not imposed. To simplify, our cost calculation does not incorporate service fees at this juncture, which could potentially lead to an underestimation of the actual costs associated with non-home-based charging events.

### Estimating carbon emissions for each charging event

Next, we focus on calculating the carbon emissions for each charging event. Instead of employing a conventional approach that involves a straightforward multiplication of energy used per charging event by the emission factor, we sought a more precise estimation of carbon emissions. This estimation should reflect the unique operating status of each vehicle post-charging event. Thus, we adopted an exact-matching method to align each charging event with its immediate subsequent driving event, according to the ending time of the charging event and the starting time of the driving event. We then calculated carbon emissions for each charging event based on the energy consumption and corresponding driving mileage of the directly subsequent driving event. This calculation method can more accurately mirror the real-world operating patterns of each vehicle and their real-time battery status. To be specific, we used the following Eq. (3) to compute carbon emissions per hundred kilometers for each charging event post-driving:

3 $$\:C=100\cdot\:\frac{{E}_{{CO}_{2}}\cdot\:\varDelta\:soc\cdot\:{B}_{capacity}}{\varDelta\:distance}$$.

In Eq. (3), *C* represents for the carbon emissions for each charging event post-driving per 100 km, $\:\varDelta\:soc$ represents the change of SOC during the directly subsequent driving event, while $\:{B}_{capacity}$ refers to the battery capacity of the BEV (kW·h), $\:\varDelta\:distance$ is the driving mileage of the BEV during the directly adjacent driving event. The electricity carbon emission factor $\:{E}_{{CO}_{2}}$ is calculated in accordance with Eq. (4).

4 $$\:{E}_{{CO}_{2}}=\frac{{T}_{E}\times\:{T}_{C}\times\:\phi\:}{{t}_{M}\times\:{i}_{ch}\times\:(1-{i}_{tr})}\:.\:\:\:\:\:\:\:\:\:\:\:\:\:\:\:\:\:\:\:\:\:\:\:\:\:\:\:\:\:\:\:\:\:$$.

Where $\:{T}_{E}$ represents the standard coal consumption for thermal power generation, with units in kg/(kW·h). In 2019 and 2020, China’s standard coal consumption for thermal power generation was 0.3064 kg/(kW·h) and 0.3055 kg/(kW·h), respectively. $\:{T}_{C}$ represents the carbon dioxide emission factor of fuel coal, with a parameter value of 3.09. $\:\phi\:$ denotes the thermal power ratio, which is the proportion of thermal power generation to the total power generation in China, as indicated in the China Energy Statistical Yearbook (Coal consumption of power supply in thermal power plants in China from 2010 to 2020);. Over 90% of China’s coal-related greenhouse gas emissions come from thermal power generation, which emits more than ten times as much greenhouse gas as other technologies such as hydropower, nuclear power, and wind power (Lin et al., 2021). According to data from the National Bureau of Statistics, in 2019 and 2020, the national power generation was 75,034.28 billion kWh and 77,790.60 billion kWh, respectively. Thermal power generation accounted for 52,201.5 billion kWh and 53,302.5 billion kWh, respectively, representing 69.57% and 71.04% of the total power generation in 2019 and 2020(China energy big data report 2021—Electric power. https://news.bjx.com.cn/html/20210617/1158638.shtml). $\:{t}_{M}$ represents the conversion factor between fuel coal and standard coal, with a parameter value of 1.07. $\:{i}_{ch}$ represents the charging efficiency, which is the ratio of the input electrical energy to the power battery from the grid. It is based on the initial charge and discharge efficiency of the BEV battery, estimated to be 98% (Yang, et al., 2018). $\:{i}_{tr}$ represents the line loss rate, which is the percentage of lost electricity during the transmission and distribution process compared to the supplied electricity. According to data published by the Chinese National Energy Administration, the comprehensive line loss rate in the power grid was 5.93% and 5.62% in 2019 and 2020, respectively.

### Optimization charging strategies based on neural network

Following this, we proceeded to construct a three-layer backpropagation neural network using the PyTorch framework. The architecture of the neural network comprises one hidden layer and one output layer. The model is trained to analyze the relationships between charging parameters (e.g., start time, initial SOC, charging speed) and their impacts on costs and emissions. The model minimizes prediction errors to ensure reliable behavioral analysis. Thanks to multiple layers of fully connected neurons, the model is capable of carrying out non-linear transformations of features, thereby enabling it to learn the complex relationships that exist between input characteristics.

In order to assess the performance of the model, the dataset is divided randomly into training and testing sets, with 80% of the samples being utilized for training and the remaining 20% reserved for testing. The training data consists of historical charging events, including variables such as initial SOC, charging start time, charging speed, battery type, and battery capacity, while the test data is used to evaluate the model’s generalization ability on unseen scenarios. The training process encompasses 100 epochs and is optimized with a batch size of 32. To determine the optimal hyperparameters, we conducted a grid search over a range of values for the learning rate (0.0001 to 0.01), batch size (16, 32, 64), and number of epochs (50 to 150). The final learning rate of 0.001 and batch size of 32 were selected based on their ability to balance convergence speed and model stability, while 100 epochs were chosen based on early stopping criteria to prevent overfitting.

During each epoch, the model iterates through the training set, processing data one batch at a time. The backpropagation algorithm is implemented to update model parameters and minimize the loss function throughout the training phase. The average loss value is recorded for each epoch and used as a monitoring metric for tracking the progress of the model’s training. To further validate the robustness of the model, we performed 10-fold cross-validation on the training set, achieving consistent performance across all folds with a mean squared error (MSE) of 1.23% on the test set. Visualization of the loss curve is accomplished through the use of graphs and charts in the research results, offering insights into the fluctuations in losses during the training period.

Upon the completion of the training phase, the model is evaluated on the testing set by calculating the mean squared error between the predicted results and the actual values. The value of the testing loss serves as an indicator of the predictive performance of the model. Multiple metrics are simulated under a variety of scenarios to optimize the start SOC and charging time, with the aim of achieving cost-saving, energy-efficient, and emission-reducing effects in ride-hailing vehicles.

#### Input layer

Given a set of charging-related features “x,” represented as an input vector.

5 $$\:X=\left[{x}_{1},{x}_{2},{x}_{3},\dots\:{x}_{n}\right]$$.

The input variables encompass the following:

The SOC at the commencement of each charging event.

The starting time for each charging event.

The maximum charging power observed in each charging event.

The charging speed associated with each charging event.

The battery power of each Battery Electric Vehicle (BEV).

The specific type of battery utilized in each BEV.

### Hidden layer

The hidden layer is realized through a fully connected layer comprising *m* neurons. Each neuron receives the entire feature vector *X* from the input layer. Through weighted linear combinations and activation functions, the hidden layer accomplishes nonlinear mappings. The output of the hidden layer can be represented as follows:

6 $$\:H=\sigma\:(X\cdot\:{W}_{hidden}+{B}_{hidden})$$.

Where:

$\:H$ denotes the output vector of the hidden layer.

$\:{W}_{hidden}$ represents the weight matrix of the hidden layer.

$\:X$ is the input vector containing charging-related features.

$\:{B}_{hidden}$ is the bias vector of the hidden layer.

$\:\sigma\:$ is the Rectified Linear Unit (ReLU) activation function, which introduces non-linearity to the model. The ReLU activation function plays a vital role in enabling the neural network to learn complex relationships between input characteristics and captures intricate patterns in the charging time prediction.

### Output layer

The output layer is also realized through a fully connected layer with one neuron. It receives the output “H” from the hidden layer as input and achieves the final prediction through a weighted linear combination. The predicted value $\:{y}_{pred}$ of the output layer can be represented as follows:

7 $$\:{y}_{pred}=H\cdot\:{W}_{output}+{B}_{output}$$.

Where:

$\:{y}_{pred}$ represents the predicted outcome, in this paper encompass the following:

The charging cost expressed in RMB per kilowatt-hour (kWh) for each charging event.

The total carbon emission quantified per 100 km for each charging event.

$\:{W}_{output}$ denotes the weight matrix of the output layer.

$\:H$ is the output vector from the hidden layer.

$\:{B}_{output}$ is the bias vector of the output layer.

### Loss function (Mean squared Error - MSE)

In this study, the mean squared error (MSE) is utilized as the loss function to measure the difference between the model’s predicted values and the true values for the training samples. For each training sample with true label *y_(true*,* i)* and model’s predicted value *y_(pred*,* i)*, the MSE can be expressed as follows:

8 $$\:MSE=\frac{1}{n}\sum\:_{i=1}^{n}{\left({y}_{true,i}-{y}_{pred,i}\right)}^{2}$$.

Where:

$\:MSE$ represents the mean squared error loss function.

$\:n$ denotes the number of training samples.

$\:{y}_{true,i}$ refers to the true value or ground truth for the *i*-th training sample, which represents the actual observed or measured value. In this study, this could refer to actual charging cost or carbon emission per charging event, depending on the specific task.

$\:{y}_{pred,i}$ represents the predicted value for the *i*-th training sample, which is the output generated by the model based on its learned parameters. This could include predicted charging cost or emissions as per the model’s output.

The Mean Squared Error (MSE) loss function serves as a pivotal metric throughout the training phase. It directs the neural network in minimizing the divergence between predicted and actual charging times, thereby progressively enhancing the predictive precision of the model.

### Optimization charging strategies based on adaptive moment Estimation algorithm

In this study, we aim to optimize the charging strategies by minimizing the MSE loss function, which quantifies the divergence between predicted charging outcomes and actual values. The optimization problem can be formally expressed as follows:

$$\:\text{min\:}L=\frac{1}{n}\sum\:_{i=1}^{n}{\left({y}_{true,i}-{y}_{pred,i}\right)}^{2}$$

Where *L* is the loss to be minimized.

n is the number of training samples.

$\:{y}_{true,i}$ refers to the true value or ground truth for the *i*-th training sample (e.g., actual charging cost or emissions).

$\:{y}_{pred,i}$ represents the predicted value for the *i*-th training sample.

The analysis framework incorporates constraints to ensure realistic parameter ranges for evaluating current charging behaviors. The first one is that the initial SOC of each vehicle must lie within a predefined range, specifically between 10% and 90%. This constraint ensures that charging occurs under optimal conditions, as charging from a very low or very high SOC can lead to inefficiencies or suboptimal charging performance. A lower SOC improves cost and carbon emission efficiency, while a higher SOC may result in less efficient charging and longer durations.

The second one is the charging speed constraint, which limits the charging power to an effective range, specifically between 20 kW and 90 kW. This constraint ensures that the charging process remains efficient while minimizing the environmental impact. Charging too fast leads to increased carbon emissions, while charging too slowly could cause operational inefficiencies.

Adam algorithm is also employed to optimize our neural network. Widely recognized in deep learning, Adam amalgamates the merits of both Root Mean Squared Propagation (RMSprop) and Momentum optimization algorithms while incorporating a bias correction feature, thereby bolstering its efficacy and stability across diverse neural network frameworks.

In the realm of deep learning, the optimization algorithms principally aim to minimize the loss function, fine-tuning the neural network’s parameters (weights and biases) to enhance performance on the training data. Adam accomplishes this by dynamically adjusting the learning rates for each parameter based on their respective gradients, permitting the learning rate to adapt to the specific attributes of different parameters. Concurrently, the algorithm leverages the concept of momentum to expedite parameter updates.

The execution of the Adam algorithm proceeds as follows:

Stage I-Initialization: Prior to commencing the training process, parameter *θ*, first moment estimate *m*, and second moment estimate *v* are initialized to zero vectors.

Stage II-Gradient Computation: At each time period *t*, the gradient $\:{g}_{t}$ of the current parameter *θ* is calculated, representing the rate of change of the loss function with respect to the parameter.

Stage III-Update First Moment Estimate *m*: Leveraging the concept of momentum, the first moment estimate $\:{m}_{t}$ of the current gradient is computed. This first moment estimate represents the exponentially weighted average of the gradients, smoothing the gradient variations.

Stage IV-Update Second Moment Estimate *v*: The second moment estimate $\:{v}_{t}$ of the current gradient is calculated. This second moment estimate represents the exponentially weighted average of the squared gradients, measuring the magnitude of gradient changes.

Stage V-Bias Correction: Since both the fist moment estimate *m* and the second moment estimate *v* are initialized as zero vectors, they can be biased at the beginning. Therefore, it is necessary to correct their biases to mitigate the influence of learning rates during the early stages of training.

Stage VI-Compute Parameter Update $\:\varDelta\:{\theta\:}_{t}$: Utilizing the corrected first moment estimate $\:{m}_{{hat}_{t}}$ and second moment estimate $\:{v}_{{hat}_{t}}$, along with the provided learning rate *α*, the parameter update $\:\varDelta\:{\theta\:}_{t}$ for the current time step is calculated.

Stage VII-Update Parameters: The parameters *θ* are then updated to new values by subtracting $\:\varDelta\:{\theta\:}_{t}$.

The Adam algorithm is expressed mathematically as follows: Given the learning rate $\:\alpha\:$ (adopting 0.001 in this research), decay rates $\:{\beta\:}_{1}$ (used for computing the first moment estimate, set to 0.9), $\:{\beta\:}_{2}$ (used for computing the second moment estimate, set to 0.999), and $\:\epsilon\:$, the Adam algorithm can be formulated as:

9 $$\:{m}_{t}={\beta\:}_{1}\cdot\:{m}_{t-1}+(1-{\beta\:}_{1})\cdot\:{g}_{t}$$

10 $$\:{v}_{t}={\beta\:}_{2}\cdot\:{v}_{t-1}+(1-{\beta\:}_{2})\cdot\:{g}_{{t}^{2}}$$

11 $$\:{m}_{{hat}_{t}}=\frac{{m}_{t}}{1-{{\beta\:}_{1}}^{t}{v}_{{hat}_{t}}}={v}_{t}/(1-\:{\beta\:}_{2}^{t})$$

12 $$\:\varDelta\:{\theta\:}_{t}=-\alpha\:\cdot\:{m}_{{hat}_{t}}/(\sqrt{{v}_{{hat}_{t}}}+\epsilon)$$

In above equations, $\:{m}_{t}$ and $\:{v}_{t}$ represent the first and second moment estimates, respectively. $\:{m}_{{hat}_{t}}$ and $\:{v}_{{hat}_{t}}$ signify the corrected first and second moment estimates, $\:\alpha\:$ denotes the learning rate, $\:{\beta\:}_{1}$ and $\:{\beta\:}_{2}$ are decay rates, $\:t$ indicates the current time step, and $\:{g}_{t}$ represents the gradient of the current parameter $\:\theta\:$.

## Electronic supplementary material

Below is the link to the electronic supplementary material.

Supplementary Material 1

## Data Availability

The raw data supporting the conclusions of this research are not publicly available due to confidentiality agreements with the data provider. However, the data underlying the figures and tables in this study are available from the corresponding author upon reasonable request.
