# Supplementary material for: A deep neural network approach for optimizing charging behavior for electric vehicle ride-hailing fleet
Source: Sci Rep. 2025 Jul 1;15:21451. doi: 10.1038/s41598-025-05953-7 (PMC12214568; doi:10.1038/s41598-025-05953-7)
Supplement: Supplementary file 1 — Supplementary Material 1 [file 41598_2025_5953_MOESM1_ESM.docx]

**Supplementary information**

**Abbreviations:**

**KNN: K-Nearest Neighbors (KNN) Algorithm**, the KNN algorithm was employed to categorize the location of charging events as either "at-home" or "otherwise." It is based on calculating distances between data points to classify them.

**HF: Haversine Formula**, a commonly used method in geodesy for measuring distances between points on a sphere, applied to calculate the distance between geographical coordinates.

**GC: Geographical Coordinates**, specified latitude and longitude values representing a location.

**HD: Haversine Distance**, the great-circle distance between two geographical coordinates, calculated using the Haversine formula.

**BT: Ball Tree Algorithm**, a data structure used in KNN algorithms for faster nearest neighbor search.

**NN: Neural Network**, a computational model composed of interconnected neurons used for non-linear function approximation and pattern recognition.

**DL: Deep Learning**, a subset of machine learning that involves the use of neural networks with multiple layers (deep neural networks) to learn complex patterns and representations from data.

**BP: Backpropagation**, an optimization algorithm used to train neural networks by updating network weights and biases based on the gradient of the loss function.

**MSE: Mean Squared Error, a** common loss function used to measure the difference between predicted values and true values in a model.

**Input Layer**: The neural network layer that receives data input.

**Hidden Layer**: The layer in a neural network located between the input and output layers, responsible for non-linear transformations and feature learning.

**Output Layer**: The final layer in a neural network responsible for producing prediction outputs.

**ReLu: Rectified Linear Unit,** a popular activation function introducing non-linearity and enhancing the expressiveness of neural networks.

**Adam: Adaptive Moment Estimation,** a widely used optimization algorithm in deep learning for efficiently optimizing neural network parameters.

**Learning Rate**: A parameter controlling the step size of parameter updates during each iteration of model training.

**Epoch**: A complete iteration of the entire dataset during the training process.

**Batch Size**: The number of samples used for updating parameters in each iteration.

**MSE Loss Function: Mean Squared Error Loss Function**, a loss function measuring the average error between predicted values and true values.

**SOC: State-of-Charge**, the current charging level of a battery.

**Carbon Emissions per Hundred Kilometers**: The measure of carbon dioxide (CO2) emissions produced by a Battery Electric Vehicle (BEV) for each hundred kilometers driven, considering different charging habits.

**Parameters:**

$\Delta lat$ is the difference in latitude between the two points. It is calculated as '$\Delta lat$ = ${lat}_{2}$-${lat}_{1}$'.

$\Delta lng$ is the difference in longitude between the two points.

${lat}_{c}$ is the latitude of the charging station.

${lat}_{r}$ is the latitude of the residential community.

$R$ is the radius of the Earth. In this paper, it is set to be 6371 kilometers.

$d$ is the haversine distance between the two points.

$E_{{CO}_{2}}$ is the electricity carbon emission factor.

$\Delta soc$ is the percentage of energy consumption of the subsequent adjacent driving event

$B_{capacity}$ is the battery capacity of the BEV (kWh)

$\Delta distance$ is the driving distance of the BEV during the adjacent driving event

$T_{E}$ is the standard coal consumption per kilowatt-hour of electricity generated by thermal power plants.

$T_{C}$ is the amount of CO2 emissions produced per unit of fuel coal used.

φ is the proportion of thermal power generation to the total power generation in China.

$t_{M}$ is the conversion factor between fuel coal and standard coal.

$i_{ch}$ is the ratio of the input electrical energy to the power battery from the grid, representing the charging efficiency of the BEV battery.

$i_{tr}$ is the percentage of lost electricity during the transmission and distribution process compared to the supplied electricity.

**Supplementary figures**

As illustrated, by progressively halving the threshold distance defining home charging from 500 meters three times, there are no significant changes in the kernel density of home-charging and non-home-charging. Nevertheless, regardless of the distance definition employed, the distributions of home charging and non-home-based charging continue to exhibit noticeable disparities.


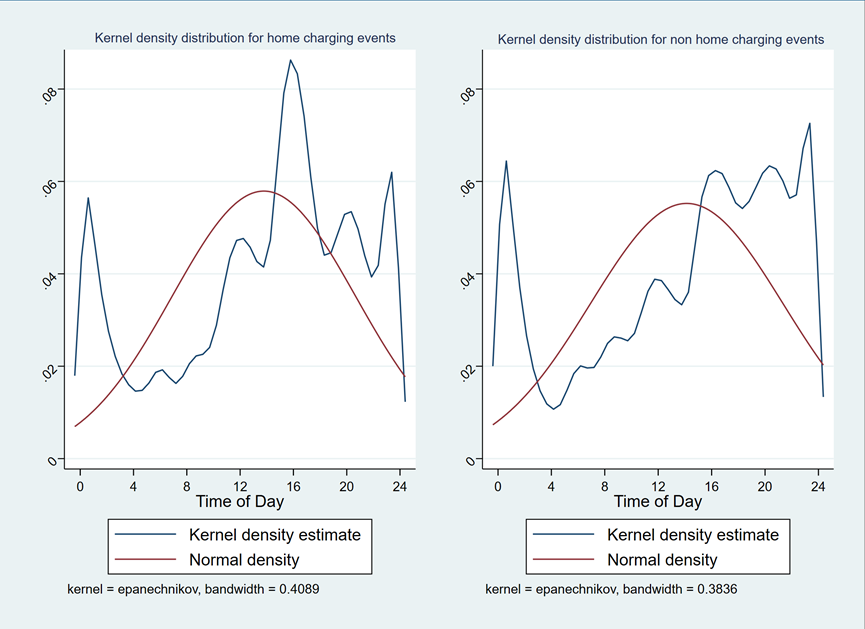


Fig. S.1. Distribution of home vs non-home-based charging based on 250 meters KNN criterion


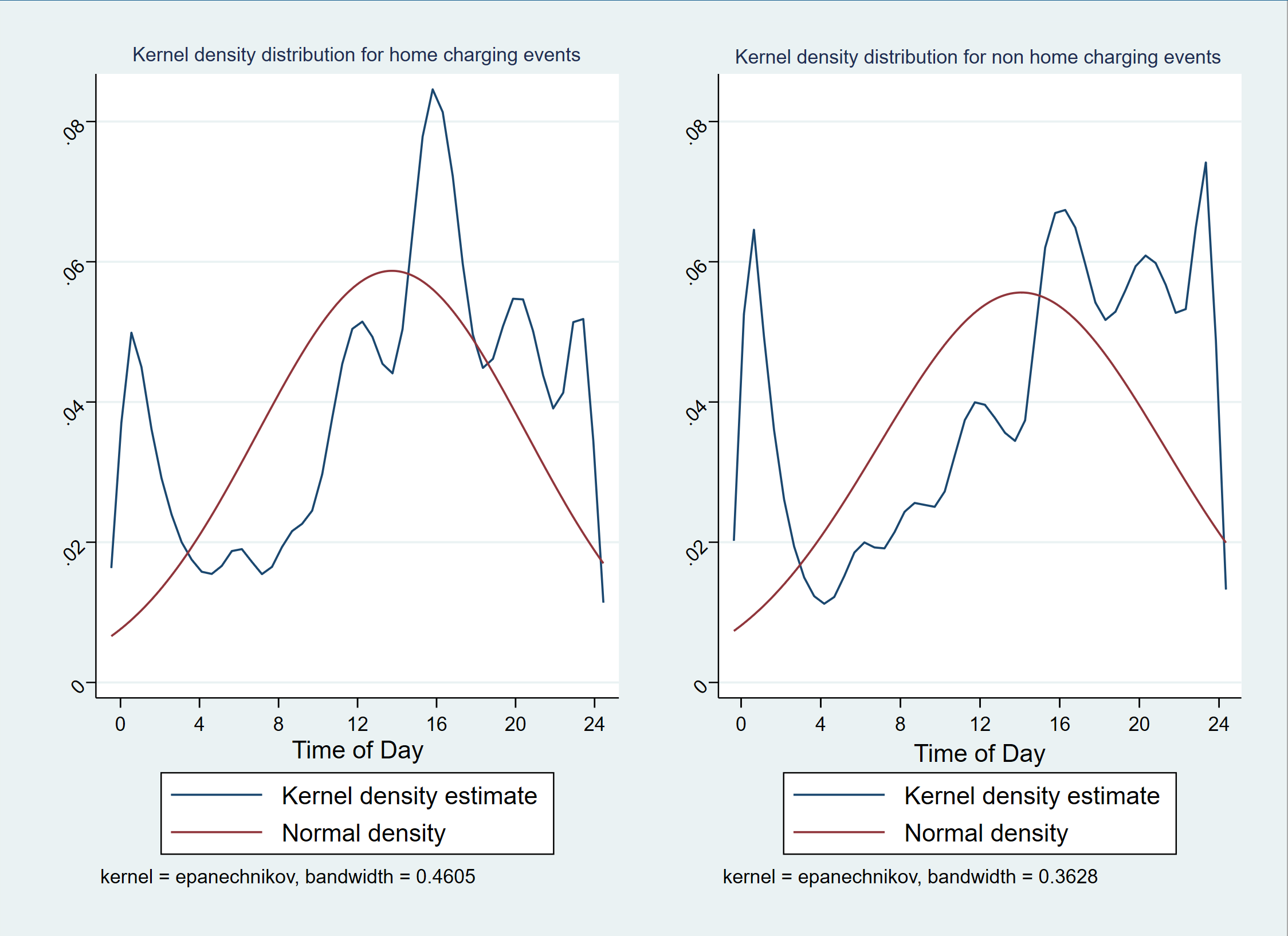


Fig. S.2. Distribution of home vs non-home-based charging based on 125 meters KNN criterion


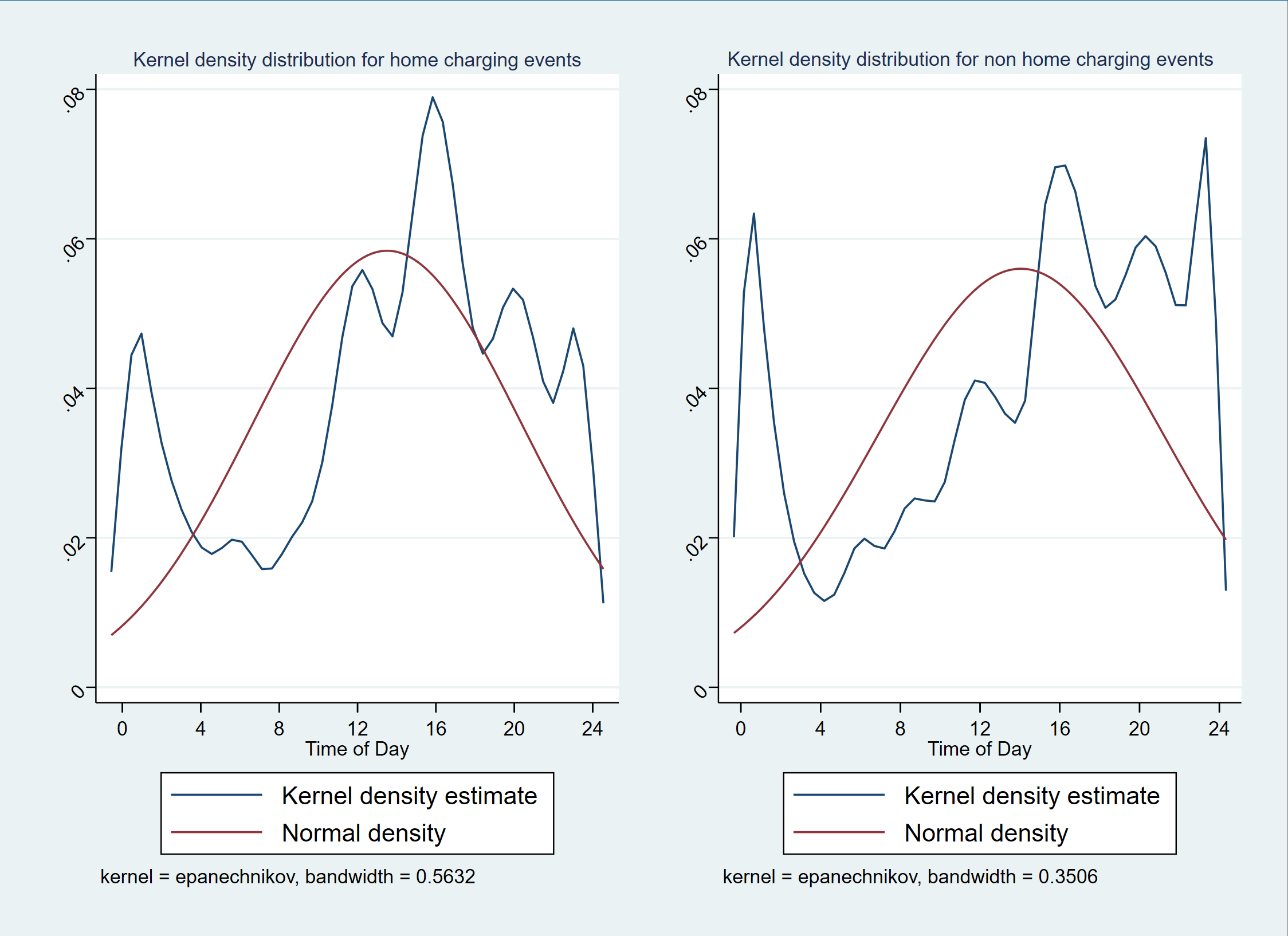


Fig. S.3. Distribution of home vs non-home-based charging based on 62.5 meters KNN criterion
